# Regulatory Impact of the C-Terminal Tail on Charge Transfer Pathways in *Drosophila* Cryptochrome

**DOI:** 10.3390/molecules25204810

**Published:** 2020-10-19

**Authors:** Martin Richter, Benjamin P. Fingerhut

**Affiliations:** Max-Born-Institut für Nichtlineare Optik und Kurzzeitspektroskopie, D-12489 Berlin, Germany; martin.richter@uni-jena.de

**Keywords:** electron transfer, circadian clock, cryptochrome, tryptophan

## Abstract

Interconnected transcriptional and translational feedback loops are at the core of the molecular mechanism of the circadian clock. Such feedback loops are synchronized to external light entrainment by the blue light photoreceptor cryptochrome (CRY) that undergoes conformational changes upon light absorption by an unknown photoexcitation mechanism. Light-induced charge transfer (CT) reactions in *Drosophila* CRY (dCRY) are investigated by state-of-the-art simulations that reveal a complex, multi-redox site nature of CT dynamics on the microscopic level. The simulations consider redox-active chromophores of the tryptophan triad (Trp triad) and further account for pathways mediated by W314 and W422 residues proximate to the C-terminal tail (CTT), thus avoiding a pre-bias to specific W-mediated CT pathways. The conducted dissipative quantum dynamics simulations employ microscopically derived model Hamiltonians and display complex and ultrafast CT dynamics on the picosecond timescale, subtly balanced by the electrostatic environment of dCRY. In silicio point mutations provide a microscopic basis for rationalizing particular CT directionality and demonstrate the degree of electrostatic control realized by a discrete set of charged amino acid residues. The predicted participation of CT states in proximity to the CTT relates the directionality of CT reactions to the spatial vicinity of a linear interaction motif. The results stress the importance of CTT directional charge transfer in addition to charge transfer via the Trp triad and call for the use of full-length CRY models including the interactions of photolyase homology region (PHR) and CTT domains.

## 1. Introduction

**Figure 1:**
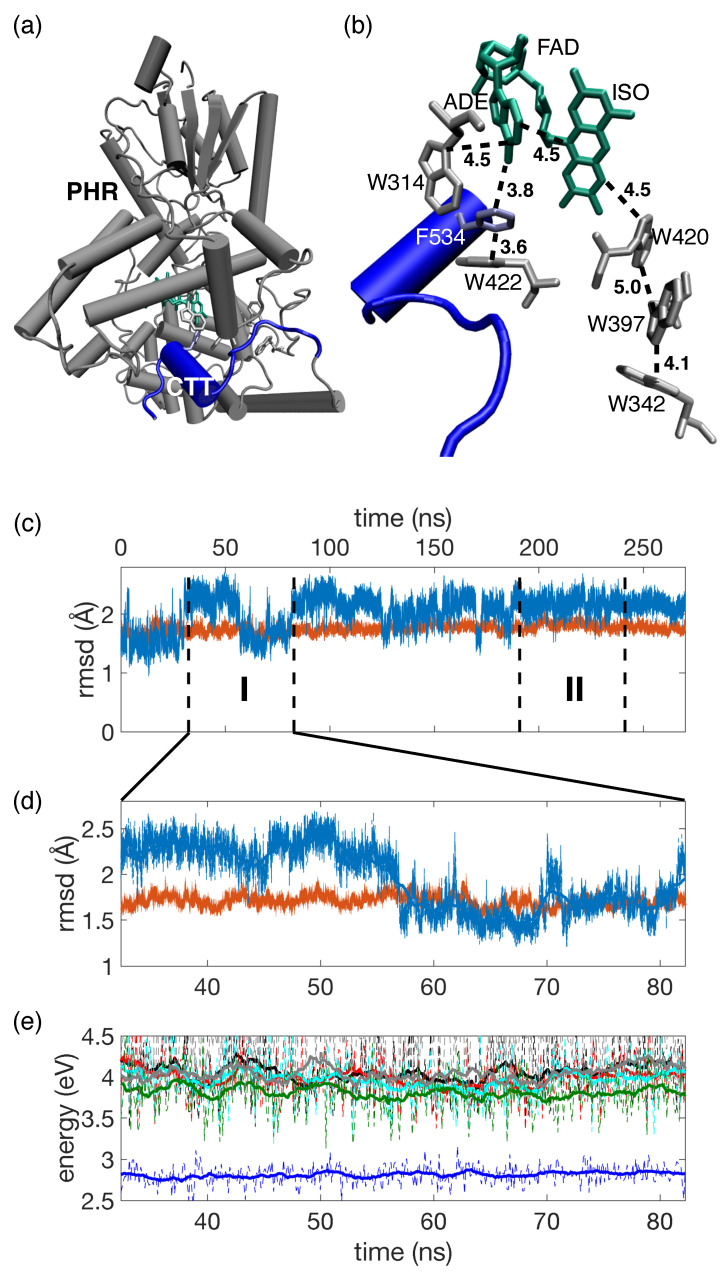
(**a**) Structure of *Drosophila* cryptochrome (dCRY), C-terminal tail (CTT) highlighted in blue (residues 520–539, PDB: 4GU5 [10]); (**b**) FAD cofactor and tryptophan residues W, edge-to-edge distances in Å; (**c**) root mean square displacements (RMSD) of dCRY (orange) and CTT (blue) of a 270 ns trajectory; I (32.4–82.8) ns and II (190.8–241.2) ns: considered time intervals in QM/MM simulations; (**d**) Zoom of RMSD during interval I; (**e**) Excitation energies of the FAD $\pi \pi^{*}$ state (blue) and CT states isoalloxazine (ISO) ${}^{-}$W420${}^{+}$ (grey), ISO${}^{-}$W397${}^{+}$ (green), ISO${}^{-}$ADE${}^{+}$ (red), ISO${}^{-}$W314${}^{+}$ (black) and ISO${}^{-}$W422${}^{+}$ (cyan) evaluated every 125 ps (dashed) on QM(TD-DFT(BNL/cc-pVDZ)/MM(*ff14SB*) level (cf. Materials and Methods, Section 4.2) together with the 2.5 ns moving average (solid).

Cryptochromes (CRYs) are highly conserved flavoproteins that share great sequence and structural homology to photolyases but lack their DNA repairing function [1,2,3]. Instead, CRYs play a central role in the regulation of the circadian cycle of bacteria, plants, and animals. In particular, the blue light photoreceptor CRY [4] synchronizes the master circadian clock to external stimuli, i.e., incident sunlight by regulating the abundance of the clock protein Timeless (TIM) via its targeting for ubiquitin-mediated degradation [5]. CRY consist of an N-terminal photolyase homology region (PHR) that binds the flavin adenine dinucleotide (FAD) cofactor, and a variable C-terminal tail (CTT) that shows high diversity in amino acid sequences among organisms [6,7,8]. X-ray structural data revealed that the CTT of *Drosophila* CRY (dCRY) forms an α-helix around an FFW motif [9,10,11] with F534 occupying the position of DNA lesion substrates binding to photolyases [12,13] (Figure 1a). As such, F534 anchors the CTT to the PHR domain. The CTT acts as repressor in the dark resting state with regulatory functionality for protein activity [14]. Upon blue light illumination CRY binds to TIM and the light-dependent recognition of TIM by dCRY involves conformational changes located in the CTT [15], as indicated by proteolytic protection assays which demonstrate that CTT exposure is increased upon light exposure [11,16].

The microscopic understanding of the photoreception mechanism and associated photochemical states of dCRY that participate in signal transduction have remained elusive and the mechanistic details of how light absorption leads to CTT conformational changes are unknown. The FAD redox state has been discussed controversially, where both oxidized FAD${}^{Ox}$ and anionic semiquinone FAD${}^{\circ -}$ have been suggested as ground states [16,17]. Correlations between photo and chemical reduction in fully oxidized flavin have been reported to be consistent with FAD${}^{Ox}$ as ground state [18]. Such light-/chemical-induced dCRY activation initiates CTT conformational changes for binding to TIM [17]. Molecular dynamics (MD) simulations suggested that protonation of H378 appears sufficient to initiate structural reorganization within the CTT, dominated by W536 translocation of the FFW motif [19]. Structurally, H378 is located between the CTT and FAD, and protonation-induced reorganization of the hydrogen bond network was proposed to induce the CTT conformational reorganization.

In analogy to tryptophan triad-dependent photoactivation in photolyases [20], it is generally assumed that photoreduction in FAD proceeds via a conserved triad of tryptophans (Figure 1b—CRY Trp triad: W420-W397-W342) [21]. In this scenario, W420 acts as primary electron donor upon photoexcitation of FAD and subsequent charge migration involves a sequence of hole (h${}^{+}$) transfer events among W residues [22,23,24,25]. Trp triad functionality was suggested as basis of CRY functionality in dCRY circadian photoreception, plant growth in Arabidopsis and magnetoreception of birds and flies [26]. Observations that dCRY promotes TIM degradation in the presence of W*n* → F mutations (*n* = 420, 397, 536), recently questioned the proposed Trp triad functionality [2]. Such observations are further corroborated by transgenic studies of magnetoreception in Trp triad-deficient *Drosophila* CRY [27] and reported physiological activity upon Trp triad mutation in *Arabidopsis* CRY1 and CRY2 [28,29] that induce photoreduction defficiency. Together these observations spurred proposals of anionic semiquinone FAD${}^{\circ -}$ as resting state [2] and alternative Y-based photoreduction pathways [30].

Here, we elucidate the mechanism of primary charge transfer (CT) in dCRY, initiated upon photoexcitation of FAD${}^{\mathrm{Ox}}$ as plausible resting state [18]. State-of-the-art simulations are performed that consider CT via the Trp triad and further take into account intra-FAD charge transfer involving the isoalloxazine (ISO) and adenine (ADE) moieties of the FAD co-factor, as well as CT states involving W314 and W422 residues (Figure 1b). The presented dissipative quantum dynamics simulations highlight the importance of CTT directional charge separation in addition to CT via the conventional Trp triad and suggest targets for site-directed mutations that allow to control CT pathways.

## 2. Results

### 2.1. Nanosecond Structural Fluctuations

X-ray structural data [9,10] reveal the spatial position of the FAD cofactor and adjacent tryptophan residues that are grouped either as belonging to the Trp triad (W420, W397, W342) or as being proximate to the CTT domain (W422, W314) (Figure 1a,b). Figure 1c presents root mean square displacements (RMSD) of dCRY for a 270 ns MD trajectory (cf. Materials and Methods, Section 4.1). For the dCRY enzyme, an RMSD < 2 Å is found with only minor deviations from the mean (orange line, Figure 1c). In contrast, the RMSD of the CTT domain shows conformational reorganization between two distinct conformational states on a time period of ≈20–30 ns around the dCRY RMSD (blue line, Figure 1c). Such modest 1–2 Å displacements demonstrate the increased flexibility of the CTT compared to the PHR domain. In particular, conformational reorganization occurs in the vicinity of the terminal residue D539 where the translocation is induced due to the formation of distinct hydrogen bonds with either G299 or R446 (SI—Figure S1).

Figure 1d shows the dCRY RMSD of a 50.4 ns trajectory segment employed in QM/MM calculations of excitation energies of the locally excited FAD $\pi \pi^{*}$ state, as well as CT states involving ISO, ADE and W420, W397, W422, W314 thereby focussing on potential primary transfer pathways (Figure 1e, cf. Materials and Methods, Section 4.2). The optical accessible FAD $\pi \pi^{*}$ state (blue), corresponding to a local excitation of the ISO moiety, shows only modest fluctuations around the mean excitation energy of 2.81 eV (441 nm, standard deviation σ = 0.12 eV). The highest level QM/MM benchmark calculations employing the ab initio LCC2 method (2.67 eV, 464 nm, SI—Table S1) provide good agreement with the first absorption band of FAD${}^{\mathrm{Ox}}$ in dCRY [16] (2.61 eV, 475 nm). The vertical excitation energies of CT states (Trp triad: ISO${}^{-}$W420${}^{+}$, ISO${}^{-}$W397${}^{+}$; CTT proximate: ISO${}^{-}$ADE${}^{+}$, ISO${}^{-}$W314${}^{+}$, ISO${}^{-}$W422${}^{+}$) are about 1 eV higher in energy and appear with comparable energetics. Fluctuations due to dCRY thermal motion are substantially larger (σ = 0.25–0.33 eV) than for the FAD $\pi \pi^{*}$ state. The comparable energetics of CT states preclude the assignment of a distinguished CT pathway, either via the Trp triad ($\pi \pi^{*}$ → ISO${}^{-}$W420${}^{+}$ → ISO${}^{-}$W397${}^{+}$) or via the alternative CTT proximate pathway ($\pi \pi^{*}$ → ISO${}^{-}$ADE${}^{+}$ → ISO${}^{-}$W314${}^{+}$/W422${}^{+}$) from vertical excitation energies only. Within numerical accuracy, the $\pi \pi^{*}$ and CT states appear independent of the the 1–2 RMSD conformational reorganization of terminal CTT residues.

### 2.2. Driving Force ΔG of Charge Transfer Reactions

**Figure 2:**
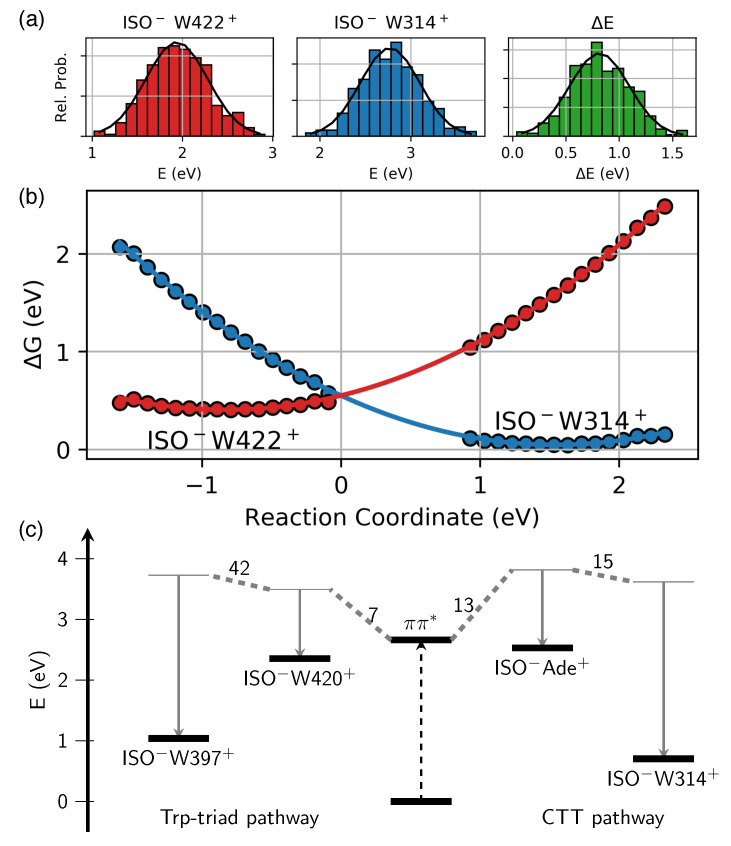
Driving forces ΔG of charge transfer (CT) reactions: (**a**) histograms of energies *E* of CT states ISO${}^{-}$W422${}^{+}$ (left) and ISO${}^{-}$W314${}^{+}$ (middle) and respective energy differences ΔE (right) for the trajectory evolving in the ISO${}^{-}$W422${}^{+}$ state. (**b**) Parabolic fit of free energy curves G(ΔE) of ISO${}^{-}$W422${}^{+}$ and ISO${}^{-}$W314${}^{+}$ states (negative and positive reaction coordinate, respectively). (**c**) ΔG and λ of CT states (LCC2-corrected, cf. Materials and Methods, Section 4.2) considered in dynamics simulations, values of interstate couplings $V_{ij}$ (in meV) as indicated.

Knowledge on reorganization energy λ, i.e., the energy required to distort the system from the reactant to the product configuration, is crucial for a determination of driving forces ΔG of CT reactions [31]. In order to account for reorganization of the anisotropic protein surrounding them, ΔG and λ of individual CT reactions have been evaluated on the QM/MM level, employing fluctuating configurations of MD trajectories that evolve in ground state equilibrium (interval I and II), as well as in respective ISO${}^{-}$WX${}^{+}$ CT states (X = 397, 420, 314, 422) (cf. Materials and Methods, Section 4.2). Figure 2a,b presents exemplary data for the CTT proximate states ISO${}^{-}$W314${}^{+}$ and ISO${}^{-}$W422${}^{+}$; the complete data set is summarized in Figure 2c and Table 1 (see also SI—Figure S2–S5).

The driving forces ΔG of primary CT reactions $\pi \pi^{*}$→ ISO${}^{-}$W420${}^{+}$ and $\pi \pi^{*}$→ ISO${}^{-}$ADE${}^{+}$ are found to be of moderate magnitude and comparable ($\Delta G^{\Delta E}$≈$\Delta G^{\mathrm{ct}}$ = −0.10–−0.16 eV, Table 1). Considering ground state equilibrium fluctuations only, both primary CT states are placed slightly above the initially excited $\pi \pi^{*}$ state ($\Delta G^{\mathrm{eq}}$ = 0.13–0.24 eV). Due to the limited sampling of configuration space of CT product states with finite length trajectories, reorganization energies λ and driving forces ΔG of the former treatment are considered more reliable [31] (cf. SI for a discussion of numerical errors).

Secondary CT states, i.e., ISO${}^{-}$W397${}^{+}$ of the Trp triad and ISO${}^{-}$W314${}^{+}$ and ISO${}^{-}$W422${}^{+}$ proximate to the CTT, are characterized by substantially larger λ due to increased solvent exposure of respective W residues, exceeding 1.5 eV due to large amplitude thermal fluctuations in CT states (cf. Table 1). Accordingly, notable driving forces ΔG≈ 0.5–1.0 eV arise for secondary CT reactions (ISO${}^{-}$W420${}^{+}$→ ISO${}^{-}$W397${}^{+}$ and ISO${}^{-}$ADE${}^{+}$→ ISO${}^{-}$W314${}^{+}$/W422${}^{+}$, respectively, Figure 2b). We find that the CT state ISO${}^{-}$W314${}^{+}$ is ≈0.3 eV below the ISO${}^{-}$W422${}^{+}$ state (Figure 2b). Compared to CT states of the Trp triad, we find ISO${}^{-}$W314${}^{+}$ slightly below the ISO${}^{-}$W397${}^{+}$ state (0.1–0.3 eV, Figure 2c, SI—Figure S5) thus forming an energetic trap of primary CT reactions.

### 2.3. Protein Electrostatic Environment

**Figure 3:**
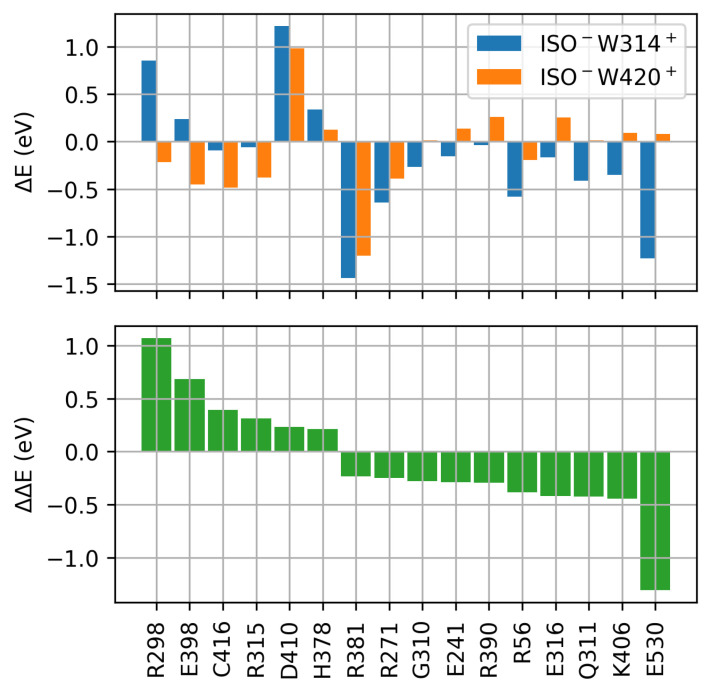
Impact of dCRY amino acid residues on CT states ISO${}^{-}$W420${}^{+}$ (Trp triad) and ISO${}^{-}$W314${}^{+}$ (CTT proximate), positive ΔE correspond to a destabilization, negative ΔE to stabiliziation of CT states (**top**). Relative stabilization/destabilization of ISO${}^{-}$W420${}^{+}$ and ISO${}^{-}$W314${}^{+}$ states (**bottom**), positive ΔΔE denote the relative stabilization of the Trp triad state ISO${}^{-}$W420${}^{+}$ while negative ΔΔE favors the CTT proximate state ISO${}^{-}$W314${}^{+}$.

The subtle energetic balance of CT states of the Trp triad and CT states proximate to the α-helical CTT domain suggests a fine tuning by the dCRY protein environment. In order to asses proteinochromic effects, a systematic evaluation of energetic shifts of CT states ISO${}^{-}$W420${}^{+}$ and ISO${}^{-}$W314${}^{+}$ induced by the electrostatic of individual amino acid residues has been performed (Figure 3 and SI—Figure S6, cf. Materials and Methods, Section 4.2 for details). In vicinity of the FAD cofactor distinct amino acid residues are identified that impose comparable stabilization/destabilization of CT states of the Trp triad and CT states proximate to the CTT domain. Due to electrostatic interaction of amino acid side chains with the ISO localized negative charge, negatively charged residues have destabilizing (ΔE>0, D410) and positively charged residues have stabilizing (ΔE<0, R381) effects, thus affecting the energy of ISO${}^{-}$W420${}^{+}$ and ISO${}^{-}$W314${}^{+}$ with equal directionality and magnitude (Figure 3, top).

Amino acid residues imposing energetic shifts of opposite direction on Trp triad and CTT proximate CT states were further identified. In particular, negatively charged amino acid E398 in vicinity of the Trp triad imposes a stabilization of the W-located hole, and thus stabilizes the ISO${}^{-}$W420${}^{+}$ CT state, compared to the ISO${}^{-}$W314${}^{+}$ state. Positively charged residues, like R298, impose the destabilization of the W-located hole particle and thus lead to a destabilization of the ISO${}^{-}$W314${}^{+}$ CT state with respect to the ISO${}^{-}$W420${}^{+}$ CT state of the Trp triad. Accordingly, E398 and R298 both favor CT states involving the Trp triad (positive ΔΔE in Figure 3, bottom). Here, the directionality of the electrostatic field due to the protein environment is aligned with h${}^{+}$ translocation from the ISO moiety towards W420 and W397. In contrast, negatively charged E530 is an integral part of the CTT and is located in the vicinity of ISO${}^{-}$W314${}^{+}$. Accordingly, the W-located h${}^{+}$ of the ISO${}^{-}$W314${}^{+}$ CT state is stabilized by E530, leading to the stabilization of CTT proximate states with respect to CT states of the Trp triad.

### 2.4. Charge Transfer Dynamics

**Figure 4:**
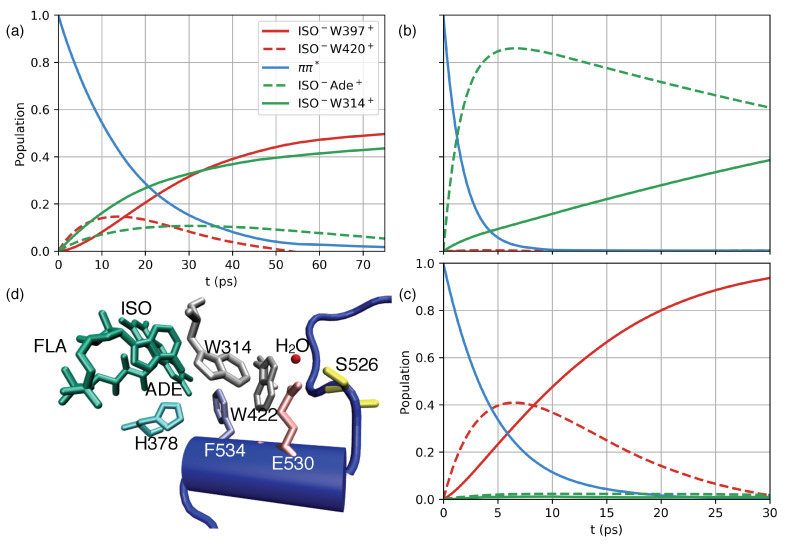
Nonequilibrium dissipative quantum dynamics of model (i)-wild-type dCRY (**a**), model (ii)-in silicio mutated R298 and E398 (**b**), and model (iii)-CTT-deficient dCRYΔ [14] mimic (**c**) constructed upon neutralization of electrostatic interaction of photolyase homology region (PHR) and CTT domains; dynamics simulations were performed with the path-integral based MACGIC-QUAPI method [32], see Materials and Methods, Section 4.4 and Section 4.5 for technical details. (**d**) Arrangement of FAD cofactor and tryptophan residues W314 and W422 together with residues of the CTT (F534, E530 and S526) and H378. The $\pi \pi^{*}$→ ISO${}^{-}$adenine (ADE)${}^{+}$→ ISO${}^{-}$W314${}^{+}$ CT pathway relates CTT directional charge transfer dynamics to spatial vicinity of linear interaction motifs comprising E530 and S526 which are integrated into a hydrogen bond network with a crystal water, see text for discussion.

The real-time CT dynamics initiated upon excitation of the $\pi \pi^{*}$ state of the FAD cofactor have been simulated by employing a quasi-adiabatic path-integral treatment [32] and QM/MM derived model Hamiltonians. In particular, CT via the Trp triad ($\pi \pi^{*}$→ ISO${}^{-}$W420${}^{+}$→ ISO${}^{-}$W397${}^{+}$), as well as an alternative CTT proximate pathway ($\pi \pi^{*}$→ ISO${}^{-}$ADE${}^{+}$→ ISO${}^{-}$W314${}^{+}$) are considered. Both pathways are characterized by comparable energetics and electronic couplings (Figure 2c and Equation (8)). Three different dCRY model Hamiltonians are constructed (Figure 4), describing (i) the wild-type CT dynamics, (ii) analyzing the impact of in silico point mutations of charged E398 and R298 residues, and (iii) assessing the influence of the CTT by mimicking dCRYΔ [14]. For the latter, the electrostatic interaction of PHR and terminal 20 amino acid residues of the CTT domain were neutralized (Figure 1, cf. Material and Methods, Section 4.4 for details).

The simulated CT dynamics of wild-type dCRY (Figure 4a) show incoherent population transfer characterized by depopulation of the initially excited $\pi \pi^{*}$ state on the 10–20 ps timescale and a parallel population of both primary CT states, i.e., ISO${}^{-}$W420${}^{+}$ and ISO${}^{-}$ADE${}^{+}$. As such, the dynamics simulations confirm the energetic picture that both pathways can potentially contribute to the deactivation of the initially excited $\pi \pi^{*}$ state of the ISO moiety. The primary CT states ISO${}^{-}$W420${}^{+}$ and ISO${}^{-}$ADE${}^{+}$ show maximum intermediate populations of ≈10–20 % after 10 and 30 ps, respectively, and are further depopulated via secondary CT states, eventually leading to a comparable population of ISO${}^{-}$W397${}^{+}$ of the Trp triad and ISO${}^{-}$W314${}^{+}$ proximate to the CTT after ≈70 ps.

The impact of dCRY electrostatic environment on CT dynamics is first investigated by in silicio mutation of amino acid residues R298 and E398 (Figure 4b). In model (ii) state energies of the Trp triad are strongly destabilized while CT states proximate to the CTT domain are stabilized due to the proximity of R298 to W314 (Figure 3). The modified energetics substantially affect the CT dynamics, showing ultrafast depopulation of the ISO $\pi \pi^{*}$ state on the sub-5 ps timescale with sequential population of the $\pi \pi^{*}$→ ISO${}^{-}$ADE${}^{+}$→ ISO${}^{-}$W314${}^{+}$ pathway. Compared to wild-type dCRY dynamics, population transfer occurs accelerated within the FAD cofactor, initially populating ISO${}^{-}$ADE${}^{+}$, followed by subsequent transfer to ISO${}^{-}$W314${}^{+}$. Notably, the moderately altered energetics of the ISO${}^{-}$ADE${}^{+}$ state (≈0.35 eV) substantially facilitate the depopulation of the $\pi \pi^{*}$ state. Such acceleration compared to the wildtype model (i) demonstrates the sensitivity of CT dynamics to subtle details of state energetics and closely resembles the ultrafast (1–2 ps) dynamics reported for insect CRY [33]. Even though CT states of the Trp triad are explicitly considered in the simulations, the respective transient population is always <2% and population of the Trp triad pathway is not observed.

The CT dynamics of model (iii), resembling dCRYΔ due to the absence of charge–charge interaction between the CTT and PHR domain, are characterized by the sole population of CT states of the Trp triad (Figure 4c, $\pi \pi^{*}$→ ISO${}^{-}$W420${}^{+}$→ ISO${}^{-}$W397${}^{+}$). The dynamics appear accelerated compared to the wild-type dCRY model (i) with depopulation of the ISO $\pi \pi^{*}$ state on the ≈7 ps timescale and the maximum transient population (≈40%) of the ISO${}^{-}$W420${}^{+}$ state after 5–7 ps. Due to the absence of favorable, i.e., stabilizing interaction with the CTT domain, in particular from negatively charged E530 (Figure 3), the CT dynamics involving state ISO${}^{-}$W314${}^{+}$ are substantially reduced (<3%). The altered electrostatic environment of model (iii) thus suppresses CTT directional charge transfer and imposes dynamics involving residues of the Trp triad only.

## 3. Discussion

The presented theoretical analysis of CT state energetics and dynamics in dCRY provides functional versatility that avoids pre-bias to specific W-mediated CT pathways and identified the microscopic impact of distinguished amino acid residues. The simulations rely on state-of-the-art first principles QM/MM methods, employing modern range separated density functionals that provide a balanced description of locally excited and charge transfer states, further benchmarked by the high level ab initio coupled cluster method LCC2. Reorganization energies λ and driving forces ΔG of CT reactions account for thermal fluctuations in dCRY in various reactant and product states where an extensive sampling of configuration space in unprecedented long time MD trajectories (0.63 μs total simulation time) provides uncorrelated snapshots for Gaussian statistics, a prerequisite for the reliable determination of CT state energetics. The time evolution of the non-equilibrium dissipative quantum dynamics is simulated with the recently introduced path integral-based MACGIC-QUAPI method [32] that shows convergence to numerical exact results for arbitrary system-environment coupling strength which is particularly relevant for CT states, characterized by appreciable λ and ΔG.

The relative energetic of CT states is found to be decisively affected by the dCRY dynamic fluctuation properties, giving rise to varying, >1 eV reorganization energies λ that reflect the non-anisotropic medium reorganization at different W sites. Comparably large values have been reported for *Arabidopsis thaliana* cryptochrome-1 (AtCry) [23,24,34]. We note that direct comparison is, to some extent, limited by differing theoretical methods, varying trajectory length, as well as the inherent differences of the electrostatic environment of plant and animal CRY (AtCry and dCRY, respectively). The particular medium reorganization of CT reactions can be rationalized on the basis of the solvent exposure of the dCRY active site. The acceptor states of secondary CT reactions (ISO${}^{-}$W397${}^{+}$ and ISO${}^{-}$W314${}^{+}$) are located in vicinity to the protein surface where, due to the increased fluctuation amplitudes of CT states, a higher local dielectric constant is suggested.

The presented approach provides an unbiased description of the W-mediated CT dynamics within dCRY and accounts for the electrostatic interaction of the CTT with the FAD-hosting active site of the PHR domain. Starting from the static information of X-ray structural data [9,10], a microscopic description of primary CT dynamics upon photoexcitation of dCRY is provided. The findings reveal the importance of CT dynamics involving ADE and W moieties proximate to the CTT (ISO${}^{-}$ADE${}^{+}$, ISO${}^{-}$W314${}^{+}$ and ISO${}^{-}$W422${}^{+}$) in addition to conventional Trp triad mediated CT and suggest that the ADE moiety actively participates in the initial steps of charge separation. As such, an active role of the U-shape of the FAD cofactor is suggested for dCRY functionality, similar to recent reports on class I/II photolyases [35]. The CTT proximate ISO${}^{-}$W314${}^{+}$ CT state appears energetically comparable to ISO${}^{-}$W397${}^{+}$, as secondary CT state of the Trp triad and thus constitutes a potential energetic trap on the ≈100 ps timescale.

The investigation of dCRY CT dynamics subject to in silicio point mutations (Figure 4, models (ii–iii)) demonstrates a crucial role of the protein electrostatic environment for the relative ordering of Trp triad and CTT proximate CT states. The analysis of energetic shifts imposed by individual amino acid residues (Figure 3) reveals the subtle balance between CT states of the Trp triad and proximate to the CTT domain, where the relative energetics is predicted to be controlled by few distinct residues. As such, charge–charge interaction with E398 and R298 was identified to favor dynamics via the Trp triad, providing an opportunity to influence particular CT pathways via site-directed mutation experiments. The mimicking of dCRYΔ [14] via the neutralization of electrostatic interaction between the CTT and PHR domains substantially alters the CT dynamics, suppressing the dynamics via CTT proximate states, thus utilizing the residues of the Trp triad only. The high density of polar and charged amino acids of the CTT, in particular E530, induces a stabilization of CT states ISO${}^{-}$W314${}^{+}$ and ISO${}^{-}$W422${}^{+}$ that become comparable in energy to ISO${}^{-}$W397${}^{+}$ of the Trp triad. Accordingly, the electrostatic interaction of residues of the CTT domain with adjacent redox-active residues can actively act as switches with the ability to modify active CT pathways within dCRY. The results stress the importance of full-length CRY models that account for the (electrostatic) interactions of PHR and CTT domains in the CRY resting state in order to predict the participation of CT pathways. We note that the dCRYΔ inspired model (iii) constructed upon charge neutralization of 20 terminal amino acid residues of the CTT here serves for the investigation of CTT electrostatic influence on photoexcitation dynamics but is not intended to mimic the dCRYΔ functionality [14]. In constitutively active dCRYΔ the PHR domain active site is accessible to protein–protein interactions, the permeability of water surrounding and the docking of molecules like ATP [23] due to the loss of the repressor motif in the resting state.

The presented results are highly relevant for the development of a microscopic picture of the initial photoexcitation mechanism of dCRY. Conformational changes in the CTT are expected to occur on ns–ms timescales, which are required to prevent futile charge recombination [36]. Protonation of the ISO moiety of the FAD cofactor by D396 was suggested for AtCry [26,37], a residue not conserved for dCRY. The presented MD trajectories evolving in various CT states of dCRY suggest that primary CT reactions and large-amplitude conformational changes are decoupled and not directly initiated by e${}^{-}$/h${}^{+}$ transfer events (SI—Figure S1). The recent results of [19] propose that protonation of H378 correlates with CTT conformational changes in the FFW sequence. Notably, H378 is located in the vicinity of the CTT proximate states ISO${}^{-}$W314${}^{+}$ and ISO${}^{-}$W422${}^{+}$ (Figure 4d). Deprotonation of W314${}^{+}$ or W422${}^{+}$ following h${}^{+}$ transfer could thus stabilize transient charge-separated states of the CTT.

Sub-nanosecond tryptophan radical de-protonation was reported for class II DNA photolyases [38], suggested to be facilitated by a protein-bound water cluster. Such a situation closely resembles the structural arrangement in the vicinity of W314 and W422, where crystal water is integrated into a hydrogen bond network comprised of E530 and S526 (Figure 4d). The functional importance of this region of the CTT was highlighted in site-directed mutation experiments [39] demonstrating that E530 → P mutation suppresses interaction with Period and TIM. The identified CT pathways involving CTT proximate states ISO${}^{-}$W314${}^{+}$ and ISO${}^{-}$W422${}^{+}$ thus relate the spatial directionality of CT reactions to the vicinity of two linear interaction motifs (CRPSNEE: residue 523–529 and EEEV: residue 528–531) that constitute a putative phosphorylation site and a class III PDZ binding segment, respectively. We speculate that disruption of the hydrogen bond network upon tryptophan radical de-protonation followed by large scale conformational change in the repressor CTT could expose the buried S526 phosphorylation site (Figure 4d).

## 4. Materials and Methods

### 4.1. Molecular Dynamics (MD)

Simulations were performed in the NPT ensemble with the PMEMD program of the AMBER14 package [40]. Periodic boundary conditions are imposed with electrostatic interactions evaluated with the particle mesh Ewald method. The initial structure of dCRY was taken from [10] (PDB: 4GU5, A-chain) employing an hff14SB force field [41]; non-standard force field parameters of the FAD${}^{Ox}$ cofactor were taken from the general AMBER force field (GAFF), while point charge parameters of FAD${}^{\cdot -}$ and W${}^{+}$ were taken from [22]. Technical details are given in SI—Section I.

### 4.2. QM/MM Simulations

Mixed quantum–classical simulations were performed by evaluating QM(TD-DFT/cc-pvdz)/MM(*ff14SB*) excitation energies with electrostatic embedding for 50.4 ns trajectory segments (I [32.4–82.8] ns and II [190.8–241.2] ns (cf. Figure 1c–e) and for respective CT state trajectories ISO${}^{-}$WX${}^{+}$ (X = 397, 420, 314, 422), accounting for enzyme thermal fluctuations in CT states [31] (cf. SI-Section II for technical details). TD-DFT calculations employed the BNL functional [42], interstate electronic couplings $V_{ij}$ were calculated with the fragment-charge difference (FCD) method [43] as implemented in Q-Chem 4.3 [44]. The FCD electronic couplings are obtained via

(1) $$\begin{array}{c} V_{ij}=\frac{\left(E_{j}-E_{i}\right)\Delta q_{ij}}{\sqrt{{\left(\Delta q_{ii}-\Delta q_{jj}\right)}^{2}+4\Delta q_{ij}^{2}}} \end{array}$$

with $E_{i/j}$ being the adiabatic state energies and the charge difference matrix of donor *D* and acceptor *A*

(2) $$\begin{array}{ccc} \Delta q_{ij} & = & q_{ij}^{D}-q_{ij}^{A} \end{array}$$

(3) $$\begin{array}{ccc} & = & \int_{r\in D}\rho_{ij}\left(r\right)dr-\int_{r\in A}\rho_{ij}\left(r\right)dr \end{array}$$

and $\rho_{ij}$ being the density operator matrix element between state |i〉 and |j〉. The FCD method has been proven robust when the investigated states are not in resonance and the charge difference is reasonably large (Δq≥0.95). Reference calculations of excitation energies were obtained at the LCC2 level [45] as implemented in Molpro 2015.1 [46,47].

The influence of the non-anisotropic protein environment on CT state energetics was evaluated systematically for surrounding 121 amino acid residues within 10 Å of the FAD cofactor. In QM/MM simulations, the point charges of a single amino acid residue were removed from the dCRY point charge field polarizing the QM region; further details are given in the SI (Section IV).

### 4.3. Reorganization Energies λ and Driving Forces ΔG of CT Reactions:

Reorganization energies λ and driving forces ΔG of CT reactions in the non-anisotropic protein environment were computed numerically with three different QM/MM approaches. In the first approach, the reorganization energy λ was computed from the variance of the energy gap fluctuations $\delta \Delta E=\Delta E-{\langle \Delta E\rangle }_{x}$ [48]

(4) $$\lambda_{x}^{\mathrm{eq}}=\frac{{\langle \delta \Delta E\cdot \delta \Delta E\rangle }_{x}}{2k_{B}T}$$

along an MD trajectory performed in electronic state *x*. Here, 〈δΔE·δΔE〉 denotes the variance.

Thermally evolving trajectories are expected to sample the configuration space of CT states infrequently [31]. We further employ CT trajectories that approximate MD in respective ISO${}^{-}$W${}^{+}$ charge-separated states (cf. Section 4.1). Respective reorganization energies $\lambda^{\mathrm{ct}}$ and free energy driving forces $\Delta G^{\mathrm{ct}}$ were calculated from the thermally averaged energy $\bar{E}$ of respective states (e.g., ISO W420 → ISO${}^{-}$W420${}^{+}$) accounting for fluctuations and relaxation in both donor and acceptor states. $\lambda^{\mathrm{ct}}$ is calculated from average energy differences in CT states according to

(5) $$\lambda^{ct}={\bar{E}}_{f,d}-{\bar{E}}_{f,a},$$

where *d*/*a* denote trajectories evolving in the donor *d* or acceptor state *a* and *i*/*f* are initial and final configuration of the CT process, respectively. Similarly, $\Delta G^{ct}$ is evaluated from the energy difference in the relaxed initial configuration evolving in the donor electronic state and relaxed final configuration evolving in the acceptor electronic state:

(6) $$\Delta G^{ct}={\bar{E}}_{i,d}-{\bar{E}}_{f,a}.$$

Reorganization energies $\lambda^{\Delta E}$ and driving forces $\Delta G^{\Delta E}$ (cf. Table 1) were additionally obtained from constructed free energy curves $G_{n}\left(\Delta E\right)$ (with *n* = i,f) of CT reactions. $G_{n}\left(\Delta E\right)$ are obtained from the energy gap probability distribution $P_{n}\left(\Delta E\right)$ sampled in MD: [23,31]

(7) $$G_{n}\left(\Delta E\right)=-RTln\left(P_{n}\left(\Delta E\right)\right)+G,$$

with temperature *T*, gas constant *R*, the energy gap ΔE of donor *d* and acceptor states *a* of the charge transfer process and *G* being an arbitrary constant (see also SI, Section III).

The long time MD trajectory data (0.63 μs total simulation time) permit extensive sampling of configuration space where statistical independent snapshots of QM/MM excitation energies obey Gaussian statistics. Further details on the evaluation of driving forces ΔG and reorganization energies λ are given in the SI (Section III).

### 4.4. Model Hamiltonian

Microscopically derived Hamiltonians of models (i–iii) (cf. Equation (8) and SI-Section V) were constructed from free energy driving forces ΔG (Table 1), corrected for systematic energy shifts compared to the LCC2 method (cf. SI—Table S1). Interstate electronic couplings $V_{ij}$ were evaluated as medians for configurations along the equilibrium trajectory (≈800 snapshots, 50.4 ns periods I and II). The Hamiltonian of wildtype dCRY model (i) in cm${}^{-1}$ is:

(8) $$H=\left(\begin{array}{ccccc} -13085 & 337 & 15 & 0 & 0\\ 337 & -2473 & 59 & 0 & 0\\ 15 & 59 & 0 & 101 & 26\\ 0 & 0 & 101 & -1127 & 117\\ 0 & 0 & 26 & 117 & -15796 \end{array}\right).$$

Hamiltonians of the in silico dCRY mutant model (ii) and CTT-deficient dCRYΔ model (iii) are given in SI Equations (S10) and (S12).

### 4.5. MACGIC-QUAPI Dynamics

The real-time non-equilibrium dynamics of model Hamiltonians (Equation (8) and SI, Equations (S10) and (S12)) were simulated with the recently developed, path integral-based MACGIC-QUAPI method [32]. Due to the employed intermediate coarse grained representation of Feynman-Vernon influence coefficients, the method provides substantial computational savings due to the reduction in considered path segments for propagation and allow us to access the regime of biologically significant long-time bath memory. The non-perturbative properties allow for simulations in arbitrary regimes of system–bath coupling strength while retaining convergence to numerically exact QUAPI results, which is particularly relevant for the large reorganization energies found for charge transfer reactions [49]. Parameter settings of the MACGIC-QUAPI simulations are given in SI, Section VI.

## 5. Conclusions

Cryptochromes posses a central role in the signal transduction of the circadian clock but the photoexcitation mechanism constitutes an unresolved mystery. Starting from the static information of X-ray structural data [9,10], we presented simulation results that rely on state-of-the-art first principles QM/MM methods and provided an unbiased description of the tryptophan-mediated CT dynamics within dCRY. The simulations fully consider the electrostatic interactions of the CTT with the FAD-hosting active site of the PHR domain. Our results reveal the active participation of the ADE moiety in the initial steps of charge separation and the importance of charge transfer states in the vicinity of the C-terminal tail. Targets for site-directed mutations that allow to control charge transfer pathways are suggested. The results stress the importance of full-length CRY models that account for the interactions of the photolyase homology region and CTT domains in order to predict active charge transfer pathways. Thus, the presented results are highly relevant for the development of a microscopic understanding of the initial photoexcitation mechanism of dCRY and for the design of bio-inspired light sensors.

## Figures and Tables

**Table 1:** Driving force ΔG and reorganization energy λ (in eV) evaluated from ground state equilibrium configurations ($\lambda^{\mathrm{eq}},\Delta G^{\mathrm{eq}}$), parabolic fitting of free energy curves ($\lambda^{\Delta E}$, $\Delta G^{\Delta E}$), and CT state configurations ($\lambda^{\mathrm{ct}},\Delta G^{\mathrm{ct}}$), cf. Materials and Methods, Section 4.2 for details, errors given in parenthesis.

State	$\lambda^{\mathrm{eq}}$	$\lambda^{\Delta E}$	$\lambda^{\mathrm{ct}}$	$\Delta G^{\mathrm{eq}}$	$\Delta G^{\Delta E}$	$\Delta G^{\mathrm{ct}}$
$\pi \pi^{*}$	0.25	—	—	—	—	—
Trp triad:					
ISO${}^{-}$W420${}^{+}$	1.10	1.14	1.14	+0.13	−0.16 (0.15)	−0.16
ISO${}^{-}$W397${}^{+}$	2.02	2.03	2.69	−0.44	−0.70 (0.31)	−1.35
CTT:						
ISO${}^{-}$ADE${}^{+}$	1.20	1.28	—	+0.24	−0.10 (0.19)	—
ISO${}^{-}$W314${}^{+}$	1.90	1.98	2.92	−0.40	−0.75 (0.22)	−1.66
ISO${}^{-}$W422${}^{+}$	1.55	1.64	1.98	−0.16	−0.52 (0.26)	−0.85

## Supplementary Materials

The following are available online. Technical details of molecular dynamics simulations and QM/MM simulations; details of evaluation of driving force of charge transfer reactions and protein environment electrostatic control; model Hamiltonians and MACGIC-QUAPI dynamics parameter settings.
