# Supplementary material for: Regulatory Impact of the C-Terminal Tail on Charge Transfer Pathways in Drosophila Cryptochrome
Source: Molecules. 2020 Oct 19;25(20):4810. doi: 10.3390/molecules25204810 (PMC7587983; doi:10.3390/molecules25204810)
Supplement: Supplementary file 1 [file molecules-25-04810-s001.pdf]

Supporting Information: Regulatory Impact of the C-Terminal Tail on Charge Transfer Pathways in Drosophila Cryptochrome

Martin Richter[1] and Benjamin P. Fingerhut[1, a)]

Max-Born-Institut für Nichtlineare Optik und Kurzzeitspektroskopie, D-12489 Berlin, Germany

(Dated: September 30, 2020)

[a)] fingerhut@mbi-berlin.de

CONTENTS

I. Molecular Dynamics Simulations 3

A. Charge transfer (CT) state trajectories 4

II. QM/MM Simulations 6

III. Driving Force ΔG of Charge Transfer Reactions 10

IV. Protein Environment Electrostatic Control 18

V. Model Hamiltonian 20

A. Wild-type dCRY 20

B. *In-silicio* dCRY mutant 21

C. CTT-deficient dCRYΔ 22

VI. MACGIC-QUAPI Dynamics: 24

A. Spectral Density: 24

B. MACGIC-QUAPI Dynamics and Convergence 27

References 29

I. MOLECULAR DYNAMICS SIMULATIONS

The initial structure of dCRY was taken from Ref. 1 (PDB: 4GU5, A-chain). Non-standard force field parameters of the FAD^{Ox} cofactor were taken from the general AMBER force field (GAFF)[2] with partial charges evaluated on the AM1-BCC level[3,4], otherwise the *ff14SB* force field[5] was used. Hydrogen atoms were added employing standard ϵ protonation state of histidines. Sodium counterions were added for charge neutrality (10 Na^+) by placing ions at positions of high electrostatic potential (*addions* command). The starting model structure was placed in a truncated octahedral solvation box with 10.0 Å buffer region employing the TIP3P water model (22245 water molecules). Divalent ion parameters of Mg^{2+} and monovalent ion parameters of Na^+ were taken from Refs. 6 and 7, respectively. Simulations have been performed with the PMEMD program of the AMBER14 package[8]. Molecular dynamics was performed in the NPT ensemble (pressure 1.0 bar, 2 ps pressure relaxation time, Langevin dynamics with 1 ps collision frequency for temperature regulation at 300 K) with a time step of 2 fs and SHAKE bond length constraints on bonds involving hydrogen atoms. Periodic boundary conditions were imposed with electrostatic interactions evaluated with the particle mesh Ewald method employing a cut-off for long range interactions of 10.0 Å. Coordinates were written to file every 250 time steps (0.5 ps).

Equilibration was performed by initial minimization of solvent and ion molecules, restraining atomic positions of dCRY (harmonic constraints 500.0 kcal/mol $Å^{-2}$, 1000 optimization steps), followed by minimization of the entire system (2500 optimization steps). Subsequent short MD (20 ps) was performed to gradually heat the system to 300 K with weak harmonic position restraints on dCRY (10 kcal/mol $Å^{-2}$), followed by 100 ps of MD without applying positions restraints in the NPT ensemble (pressure 1 bar) for density equilibration. The first 3.6 ns of the production run (NPT ensemble) were further discarded as equilibration period. Subsequent production runs cover in total 270 ns = 0.27 μs of simulation time. Geometries for the evaluation of QM/MM excitation energies (see below) during 50.4 ns periods [32.4 - 82.8] ns and [190.8 - 241.2] ns were taken every 125 ps. Post-processing of MD trajectory has been performed with the CPPTRAJ program.[9] Advanced cluster analysis with DB-SCAN clustering algorithm[10] was performed to obtain representative geometries employed for the evaluation of proteinochromic shifts induced by discrete amino acid residues (cf. SI - Sec. IV).

A. Charge transfer (CT) state trajectories

MD trajectories evolving in charge transfer (CT) states ISO^-W397^+, ISO^-W420^+, ISO^-W314^+ and ISO^-W422^+, respectively, were evaluated by a sudden change of force field parameters of the equilibration ground state trajectory at simulation time 39.6 ns (dashed dotted vertical lines in Fig. S1) thus accounting for partial charges of respective CT state. CT MD trajectories were performed in order to approximately sample the configurations in vicinity of respective ISO^-W^+ charge separated states and are further employed in QM/MM based evaluation of driving forces of CT reactions (cf. Sec. II and III)

Modified force field parameters of $FAD^{\cdot -}$ and W^+ were taken from Ref. 11 and equilibration to the new charge distribution was considered during subsequent 3.6 ns. Production MD simulations cover 90.0 ns in respective CT state amounting to 360.0 ns of simulation time (0.630 μs total simulation time). Root mean square displacements (rmsd) of dCRY evolving in respective CT state are given in Fig. S1. Following the equilibration period, geometries for the evaluation of QM/MM excitation energies were taken every 125 ps during a 50.4 ns simulation period (cf. Table S2).

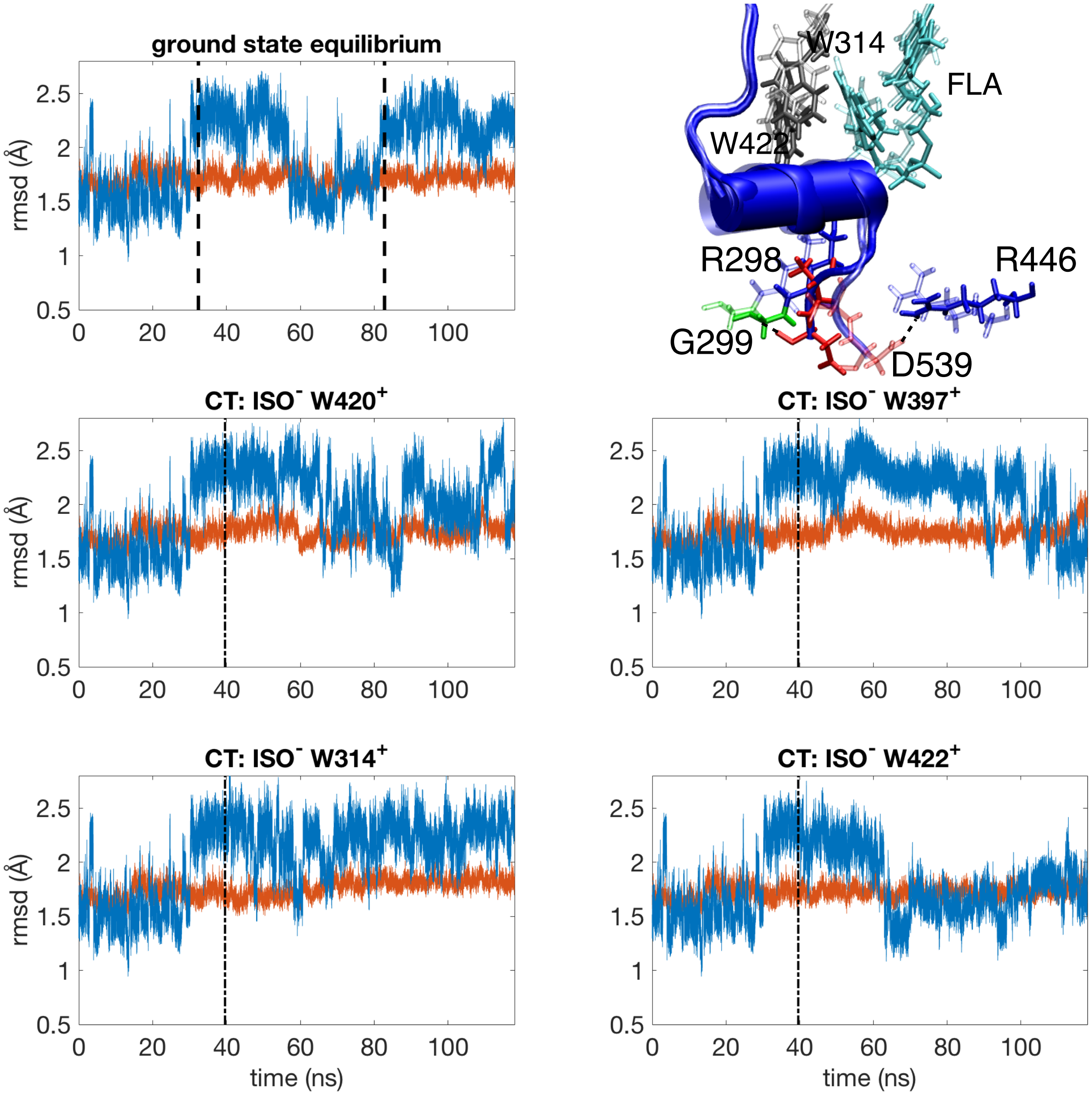

Figure S1. RMSD of dCRY (orange) and CTT (blue, residues 520-539) of the 270 ns ground state equilibrium trajectory (top left) and CT trajectories as indicated, vertical dashed lines indicate 50.4 ns period I [32.4 - 82.8] ns, dashed dotted lines mark the sudden switch of point charge parameters for charged $FAD^{\cdot -}$ and WX^{+} residues ($X = 420, 397, 314, 422$); top right: superimposed structures of most populated cluster (solid) and second most populated cluster (transparent) derived from advanced cluster analysis. RMSD of the CTT of both cluster structures is 2.05 $\AA$ dominated by translocation of terminal residue D539 which forms distinct hydrogen bonds to G299 (solid) or R446 (transparent).

II. QM/MM SIMULATIONS

Mixed quantum mechanical - molecular mechanical (QM/MM) simulations were employed for extensive sampling of configurations generated by MD simulations. QM/MM simulations were performed every 125 ps of 50.4 ns trajectory segments for trajectories sampling ground state equilibrium configurations (I [32.4 - 82.8] ns and II [190.8 - 241.2] ns, cf. Fig. 1c of main text) and for trajectories evolving in CT states ISO^-W397^+, ISO^-W420^+, ISO^-W314^+ and ISO^-W422^+, respectively (cf. Table S2 for a summary of performed QM/MM simulations). For the QM region density functional theory (Basis: cc-pVDZ) with electrostatic embedding in the surrounding *ff14SB* point charge field was employed, excitation energies of locally excited states and CT states were evaluated on TD-DFT level of theory for the QM region, further benchmarked by linear-response local second-order coupled-cluster simulations (see below). The QM(TD-DFT)/MM(*ff14SB*) production run calculations of excitation energies consider 32 singlet states employing the Baer-Neuhauser-Livshits (BNL) functional[12,13] that is based on a range separation of the Coulomb operator for improved description of CT states. The range separation parameter ω of BNL functional was tuned[14,15] to gas phase excitation energy of the isoalloxazine (ISO) moiety of FAD capped by a methyl group ($\omega = 0.238$). TD-DFT simulations were performed with the QChem 4.3 program package[16].

The QM region included the ISO moiety together with two additional aromatic amino acid side chains that are systematically varied to obtain the necessary state energies and interstate couplings (e.g. ISO - ADE - W314, ISO - W420 - W397, cf. Table S2). Bonds crossing the QM/MM boundary were truncated with hydrogen atoms in the QM region with respective bond lengths scaled by 0.709. Corresponding classical atoms of the MM region are deleted to avoid overpolarization. Assignment of state character was performed with the fragment-charge difference (FCD) method[17] where a charge difference threshold of 0.90 was used to identify CT states, and optical accessible $\pi\pi^*$ states were identified with a threshold on oscillator strength > 0.05.

a. TD-DFT Benchmark Simulations: QM(TD-DFT)/MM(*ff14SB*) calculations were further benchmarked by ab-initio linear-response local second-order coupled-cluster simulations for excited states (LCC2)[18–21] that inherently provides a balanced description of locally excited and CT states. We consider electrostatic embedding within the surrounding *ff14SB*

point charge field (QM(LCC2)/MM(*ff14SB*)) employing Molpro program package[22,23]. For comparison we consider the BNL, as well as popular ωB97X-D and CAM-B3LYP functionals (Table S1). QM(LCC2)/MM(*ff14SB*) reference calculations were performed for 30 snapshots of dCRY and 30 snapshots of *in silicio* mutated dCRY (charge neutralized R298 and E398, see Sec. V B) of trajectory segment I ([32.4 - 82.8] ns). Due to convergence difficulties of the large scale LCC2 simulations (QM region ISO, ADE, W314: 700 basis functions, $1.01 \cdot 10^7$ CSFs; QM region ISO, W420, W397: 696 basis functions, $1.04 \cdot 10^7$ CSFs) $\approx$ 50 % of LCC2 simulations provided converged results (cf. Table S1).

For the reference QM(LCC2)/MM(*ff14SB*) simulations we find good agreement of $\pi\pi^*$ excitation energy with the first absorption band of dCRY (Exp: 2.61 eV, 475 nm;[24] QM(LCC2)/MM(*ff14SB*): 2.65-2.69 eV, 464 nm, Table S1). For the tuned BNL functional we find a balanced description of ISO-localized $\pi\pi^*$ states of FAD and CT states involving the ADE moiety (error $\approx$ +0.14 and +0.15 eV, respectively) while for CT states involving W moieties the discrepancy to the reference is somewhat larger (error $\approx$ +0.29-0.45 eV). For comparison, CAM-B3LYP systematically overestimates the energy of $\pi\pi^*$ states and underestimates CT state energies imposing larger errors on relative energetics of $\pi\pi^*$ and CT states. The performance of the ωB97X-D functional is comparable to BNL where CT state energies are overestimated by +0.3-0.4 eV and $\pi\pi^*$ state energy by about 0.25 eV.

Relative energy shifts ΔE of locally excited $\pi\pi^*$ and CT states (last column Table S1) have been employed in the construction of model Hamiltonians (see. Sec. VI A), thus accounting for the systematic error of QM(TD-DFT(BNL))/MM(*ff14SB*) with respect to QM(LCC2)/MM(*ff14SB*) method.

Table S1. Averaged excitation energies (number of samples # as indicated) of the QM(LCC2)/MM(*ff14SB*)= *LCC2* reference method and QM(TD-DFT)/MM(*ff14SB*) methods, employing *BNL* (top), *CAM-B3LYP* (middle, C-B3LYP) and *ωB97X-D* density functionals, together with relative deviations ΔE; Energies in eV.

	wildtype				mutant				total			
State	#	*BNL*	*LCC2*	ΔE	#	*BNL*	*LCC2*	ΔE	#	*BNL*	*LCC2*	ΔE
ISO^-W397^+	10	4.066	3.685	0.381	7	4.945	4.484	0.461	17	4.428	4.014	0.414
ISO^-W420^+	10	3.847	3.568	0.279	10	4.336	4.031	0.305	20	4.091	3.799	0.292
$\pi\pi^*$ (ISO)	32	2.822	2.668	0.154	23	2.793	2.665	0.128	55	2.810	2.667	0.143
ISO^-ADE^+	22	3.936	3.765	0.171	13	3.459	3.256	0.203	35	3.759	3.576	0.183
ISO^-W314^+	22	4.063	3.604	0.459	13	3.280	2.863	0.417	35	3.772	3.329	0.443
State	#	*C-B3LYP*	*LCC2*	ΔE	#	*C-B3LYP*	*LCC2*	ΔE	#	*C-B3LYP*	*LCC2*	ΔE
ISO^-W397^+	10	3.313	3.685	-0.371	7	4.214	4.484	-0.270	17	3.684	4.014	-0.330
ISO^-W420^+	10	3.428	3.568	-0.140	10	3.907	4.031	-0.124	20	3.668	3.799	-0.132
$\pi\pi^*$ (ISO)	31	2.955	2.676	0.279	22	2.929	2.623	0.306	53	2.944	2.654	0.290
ISO^-ADE^+	21	3.694	3.789	-0.095	12	3.182	3.285	-0.103	33	3.508	3.606	-0.098
ISO^-W314^+	21	3.353	3.629	-0.276	12	2.555	2.811	-0.256	33	3.063	3.332	-0.269
State	#	*ωB97X-D*	*LCC2*	ΔE	#	*ωB97X-D*	*LCC2*	ΔE	#	*ωB97X-D*	*LCC2*	
ISO^-W397^+	9	4.055	3.711	0.343	6	4.947	4.482	0.464	15	4.411	4.020	0.392
ISO^-W420^+	9	3.876	3.565	0.311	9	4.371	4.050	0.320	18	4.123	3.808	0.316
$\pi\pi^*$ (ISO)	22	2.954	2.706	0.248	22	2.920	2.674	0.246	44	2.937	2.690	0.247
ISO^-ADE^+	14	4.106	3.807	0.299	13	3.556	3.256	0.300	27	3.841	3.541	0.300
ISO^-W314^+	14	4.043	3.660	0.383	13	3.277	2.863	0.414	27	3.675	3.276	0.398

Table S2. Summary of QM(TD-DFT(BNL))/MM(*ff14SB*) production run calculations performed for configurations of ground state equilibrium trajectories (I [32.4 - 82.8] ns and II [190.8 - 241.2] ns) and for configurations of trajectories evolving in CT states $ISO^{-}W397^{+}$, $ISO^{-}W420^{+}$, $ISO^{-}W314^{+}$ and $ISO^{-}W422^{+}$, respectively. For details on (R298 / E398 → 0) mutant and dCRYΔ see Secs. IV, V B and V C.

Trajectory	MM environment	QM region	# snapshots
GS (I, 40 ps)	wildtype	ISO, ADE, W422	4000
GS (I)	wildtype	ISO, ADE, W314	399
GS (I)	wildtype	ISO, W314, W422	398
GS (I)	wildtype	ISO, W420, W397	399
GS (I)	wildtype	ISO, W397, W342	398
GS (II)	wildtype	ISO, ADE, W314	400
GS (II)	wildtype	ISO, W314, W422	395
GS (II)	wildtype	ISO, W420, W397	400
GS (II)	wildtype	ISO, W397, W342	395
GS (I)	(R298 / E398 → 0) mutant	ISO, ADE, W314	400
GS (I)	(R298 / E398 → 0) mutant	ISO, W314, W422	400
GS (I)	(R298 / E398 → 0) mutant	ISO, W420, W397	400
GS (I)	dCRYΔ	ISO, ADE, W314	395
GS (I)	dCRYΔ	ISO, W420, W397	395
$ISO^{-}W397^{+}$	wildtype	ISO, W314, W422	395
$ISO^{-}W397^{+}$	wildtype	ISO, W420, W397	395
$ISO^{-}W420^{+}$	wildtype	ISO, W420, W397	401
$ISO^{-}W314^{+}$	wildtype	ISO, ADE, W314	401
$ISO^{-}W314^{+}$	wildtype	ISO, W314, W422	401
$ISO^{-}W314^{+}$	wildtype	ISO, W420, W397	401
$ISO^{-}W422^{+}$	wildtype	ISO, W314, W422	401
		total:	11969

III. DRIVING FORCE ΔG OF CHARGE TRANSFER REACTIONS

Reorganization energy λ was computed from the variance of the energy gap fluctuations $\delta\Delta E = \Delta E - \langle\Delta E\rangle_x$[25]

$$\lambda_x^{\mathrm{eq}} = \frac{\langle\delta\Delta E \cdot \delta\Delta E\rangle_x}{2k_{\mathrm{B}}T} \tag{1}$$

along an MD trajectory performed in electronic state x. Here, $\langle\delta\Delta E \cdot \delta\Delta E\rangle$ denotes the variance. λ^{eq} was approximated by respective time evolution in the electronic ground state which is particularly justified for local $\pi\pi^*$ excitation of the ISO moiety due to moderate nuclear and electronic displacements.[11] Employing 799 QM(TD-DFT(BNL))/MM(*ff14SB*) snapshots (cf. Table S2), calculated reorganization energies are 0.25 eV for the $\pi\pi^*$ and 1.20 eV for the ISO^-ADE^+ CT state (Table 1 of main text).

Thermally evolving trajectories are expected to sample the configuration space of CT states infrequently.[26] We further employ CT trajectories that approximate MD in respective ISO^-W^+ charge separated state (cf. Sec. I A). Respective reorganization energies λ^{ct} and free energy driving forces ΔG^{ct} were calculated from the thermally averaged energy $\bar{E}$ of respective states (e.g. ISO W420 $\rightarrow$ ISO^-W420^+) accounting for fluctuations and relaxation in both, donor and acceptor states. λ^{ct} is calculated from average energy differences of CT states according to

$$\lambda^{ct} = \bar{E}_{f,d} - \bar{E}_{f,a}. \tag{2}$$

Here, d/a denote trajectories evolving in the donor d or acceptor state a and i/f are initial and final configuration of the CT process, respectively. Again, time evolution of the ISO $\pi\pi^*$ excited state was approximated using the ground state equilibrium trajectory. Similarly, ΔG^{ct} is evaluated from the energy difference of the relaxed initial configuration evolving in donor electronic state and relaxed final configuration evolving in acceptor electronic state:

$$\Delta G^{ct} = \bar{E}_{i,d} - \bar{E}_{f,a}. \tag{3}$$

Thermally averaged energies $\bar{E}$ are evaluated for respective CT state, the number of employed QM(TD-DFT(BNL))/MM(*ff14SB*) snapshots is summarized in Table S2, respective distributions are given in Figs. S2-S3.

Free energy curves $G_n(\Delta E)$ with $n = i, f$ were constructed from the energy gap probability distribution $P_n(\Delta E)$ sampled in MD:[26,27]

$$G_n(\Delta E) = -RTln(P_n(\Delta E)) + G, \tag{4}$$

with temperature T, gas constant R, the energy gap ΔE of donor d and acceptor states a of the charge transfer process and G being an arbitrary constant. By defining ΔE as the reaction coordinate and setting G to zero (interest on relative energies only), the free energy of the final state $G_f(\Delta E)$ is given by

$$G_f(\Delta E) = G_i(\Delta E) + \Delta E \tag{5}$$

and the activation free energy being defined as

$$\Delta G^{\ddagger} = G_i(0). \tag{6}$$

The reorganization energy is given by

$$\lambda^{\Delta E} = G_i(\Delta E_{\mathrm{min,f}}). \tag{7}$$

and $\Delta G^{\Delta E}$ is defined by the difference of minima of both parabola. Here, $\Delta E_{\mathrm{min,f}}$ denotes the energy gap for which G_f is minimal. Again, the time evolution in the ISO $\pi\pi^*$ excited state state was approximated using the ground state equilibrium trajectory and QM(TD-DFT(BNL))/MM(*ff14SB*) snapshots that sample configurations of CT states $\mathrm{ISO^-W397^+}$, $\mathrm{ISO^-W420^+}$, $\mathrm{ISO^-W314^+}$ and $\mathrm{ISO^-W422^+}$, respectively, were employed for the constructed free energy curves (cf. Table S2 and Fig. S2-S5).

A further way to compute the reorganization energy λ is given by an integration of the spectral density, the approach is detailed in Sec. VI A

a. Relative CT state energetics: Transitions between different CT states (e.g. $\mathrm{ISO^-W397^+W314} \rightarrow \mathrm{ISO^-W397W314^+}$) were exploited to reduce the uncertainty of relative CT energetics of $\mathrm{ISO^-W397^+}$, $\mathrm{ISO^-W314^+}$ and $\mathrm{ISO^-W422^+}$ states (Fig. S5). In respective CT self exchange reactions, the different CT trajectories provide a balanced sampling of conformational space of both, reactant and product states. Accordingly, minima of reactant and product harmonic free energy curves $G_{i/f}(\Delta E)$ can be fit simultaneously, resulting in a well-defined description of relative free energy differences ΔG. Parabolic fits of free energy curves $G_{i/f}(\Delta E)$ were performed with the energetic shift along the reaction coordinate, curvature and relative energetics ΔG as free parameters. Figure S5 presents the QM/MM results of charge transfer reactions $\mathrm{ISO^-W422^+W314} \rightarrow \mathrm{ISO^-W422W314^+}$ and $\mathrm{ISO^-W397^+W314} \rightarrow \mathrm{ISO^-W397W314^+}$, together with the parabolic fits of free energy curves $G_{i/f}(\Delta E)$.

b. Discussion of CT energetics: Reorganization energies λ and free energy driving forces ΔG evaluated with the different approaches (see above) are summarized in Table 1 of the main text. Considering CT reactions $\pi\pi^* \rightarrow$ ISO$^-$W422$^+$ and $\pi\pi^* \rightarrow$ ISO$^-$W420$^+$ all employed methods provide consistent results. For CT reaction $\pi\pi^* \rightarrow$ ISO$^-$W314$^+$ and $\pi\pi^* \rightarrow$ ISO$^-$W397$^+$ the results of λ^{eq} and λ^{ct} agree qualitatively but differ in magnitude ($\approx$ 0.6 - 1 eV). Due to limited sampling of CT state configuration space with the equilibrium trajectory (λ^{eq}) we consider λ^{ct} more reliable, as sampling of configuration space of the product CT state is taken into account.

Employing bi-parabolic fits of free energy curves $G_n(\Delta E)$ for the CT reaction ISO$^-$W314$^+$W422 $\rightarrow$ ISO$^-$W314W422$^+$ yields a driving force $\Delta G = +0.34$ eV (Fig. S5, top) that is consistent with respective values derived from mono-parabolic fits of the forward reaction ($\Delta G = +0.26$ eV; ISO$^-$W314$^+$W422 $\rightarrow$ ISO$^-$W314W422$^+$). For comparison, using thermally averaged energies $\bar{E}_{i/f,d/a}$ of respective CT trajectories (Eqs. 2-3) of redox pair ISO$^-$W422$^+$W314 $\rightarrow$ ISO$^-$W422W314$^+$ free energy ΔG is found to be +0.81 eV, consistently favoring the ISO$^-$W314$^+$ state.

Respective analysis for redox couple ISO $^-$W314$^+$W397 $\rightarrow$ ISO$^-$W314W397$^+$ (Fig. S5, bottom) yields a free energy driving force $\Delta G \approx +0.10$ eV with state ISO$^-$W314$^+$W397 being slightly more stabilized by the environment. Such values are in agreement with the thermally averaged energy treatment relying on CT trajectories $\bar{E}_{i/f,d/a}$ (Eqs. 2-3) where again ISO$^-$W314$^+$W397 is found to be more stable than ISO$^-$W314W397$^+$. Employing the fitted free energy curves $G_i(\Delta E)$ (Eqs. 4-5) for transitions for ISO $\pi\pi^* \rightarrow$ISO$^-$W397$^+$ and ISO $\pi\pi^* \rightarrow$ ISO$^-$W314$^+$, respectively, yields ISO$^-$W314$^+$ about 0.05 eV below the ISO$^-$W397$^+$ state (see Table 1 of main text), although within 0.2-0.3 eV error estimates (see below).

c. Discussion of numerical errors and convergence: The long time MD trajectory data (0.63 μs total simulation time) permit extensive sampling of configuration space in vicinity of the ground state equilibrium and of CT states, and the respective statistical independent snapshots allow for Gaussian statistics of QM/MM excitation energies (Fig. S2-S3). Absolute energetic assignment of CT states is challenging due to comparable energetics, error-estimates of driving forces ΔG and the required TD-DFT treatment in QM/MM simulations. Therefore different numerical QM/MM approaches were employed to provide a consistent picture of relative energetic assignment of CT states (see above).

Methods that rely on a sampling of ground state equilibrium configuration space (Eq. 1) yield lower reorganization energies (driving forces) compared to methods that take into account the evolution in product CT states (λ^{ct} and ΔG^{ct}, respectively; Eq. 2-3). For both approaches $\approx$ 400 snapshots are sufficient for converged results ($\approx$ 0.1-0.15 eV accuracy, deviation of Gaussian fit to numerical histogram, cf. Fig S2-S3). For the constructed free energy curves (Eq. 4-7), substantially more QM/MM snapshots ($\approx$ 800) are required to reasonably reduce statistical error estimates (0.15-0.19 eV for ISO proximate CT states $\text{ISO}^{-}\text{W420}^{+}$ and $\text{ISO}^{-}\text{ADE}^{+}$; 0.22-0.31 eV for CT states $\text{ISO}^{-}\text{W397}^{+}$, $\text{ISO}^{-}\text{W314}^{+}$ and $\text{ISO}^{-}\text{W422}^{+}$). Nevertheless, by combining the evolution of trajectories in different CT states the uncertainty on relative CT state energetics can be substantially reduced in the bi-parabolic treatment ($\approx$ 0.1 eV). An inherent assumption in these treatments is $\lambda_{in} << \lambda_{out}$, i.e., that inner-sphere intramolecular reorganization, e.g. due to bond length alteration, can be neglected compared to outer-sphere medium reorganization of CT states, in agreement with findings of Ref. 11.

Extensive sampling of configuration space is facilitated only with TD-DFT methods due to the large considered QM region ($\approx$ 700 basis functions). Performance of the employed BNL functional was investigated in reference calculations against the rigorous LCC2 level of theory with relative deviation of 0.14-0.46 eV for locally excited $\pi\pi^{*}$ and CT states, respetively (Table S1). Such systematic errors were accounted for in the construction of the Hamiltonian employed for MACGIC-QUAPI dynamics simulations (cf. Sec. V).

We conclude that error margins due to methodological limitations and statistical restrictions are on the 0.2-0.3 eV order and comparable in magnitude. Systematic improvement via e.g. explicitly considering nuclear dynamics in electronic excited states and employment of high level (e.g. CASPT2) methods is in principle possible but extremely challenging. As such, we consider the presented treatment as state-of-the-art with todays computational resources and methods.

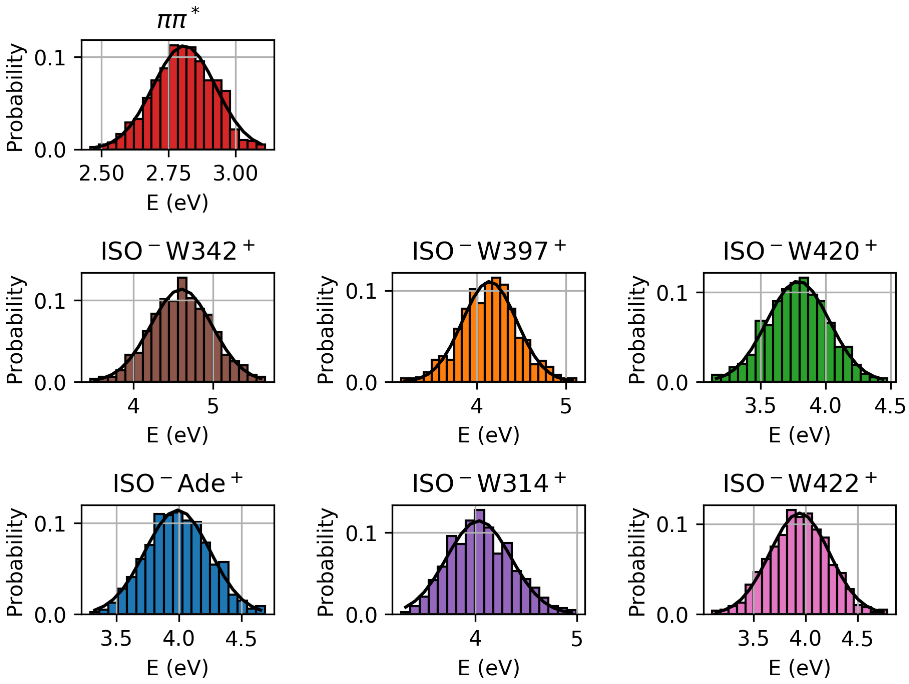

Figure S2. Histograms of excitations energies of indicated electronic states together with Gaussian fits evaluated for configurations of intervals I [32.4 - 82.8] ns and II [190.8 - 241.2] ns of a ground state equilibrium MD trajectory on QM(TD-DFT(BNL))/MM(ff14SB) level of theory ($\approx$ 800 snapshots, cf. Table S2).

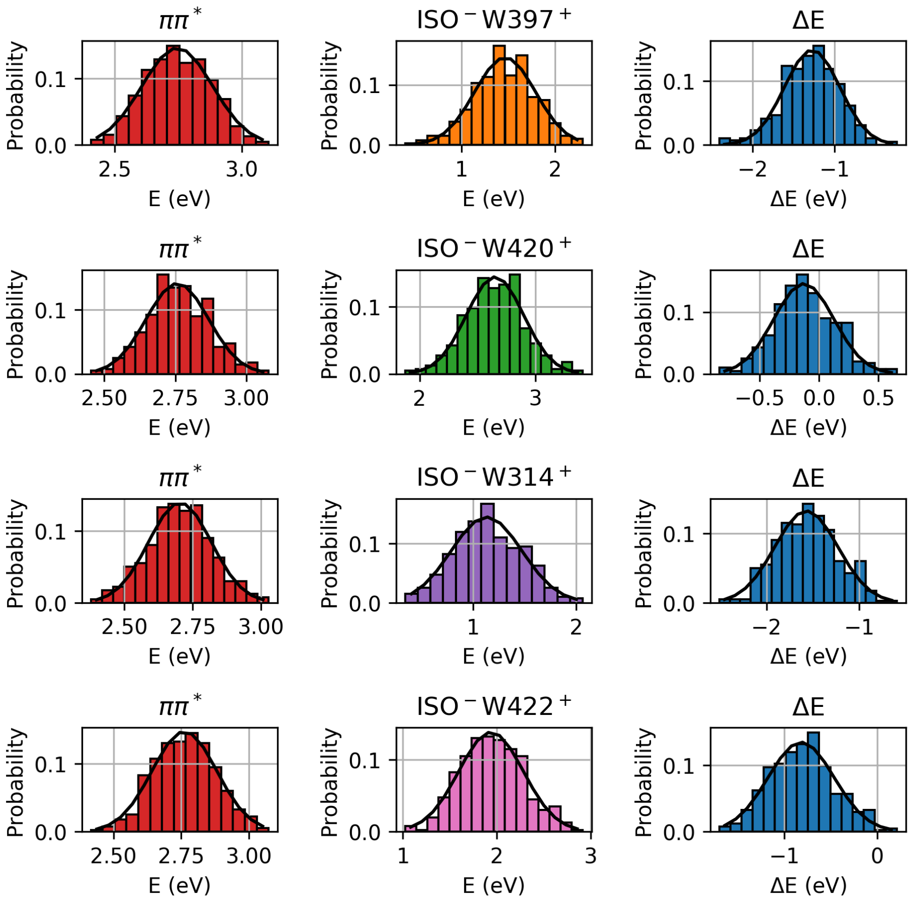

Figure S3. Histograms of excitations energies of indicated electronic states together with energy differences ΔE (right column) and Gaussian fits for configurations of MD trajectories evolving in respective CT states evaluated on QM(TD-DFT(BNL))/MM(ff14SB) level of theory ($\approx$ 400 snapshots, cf. Table S2).

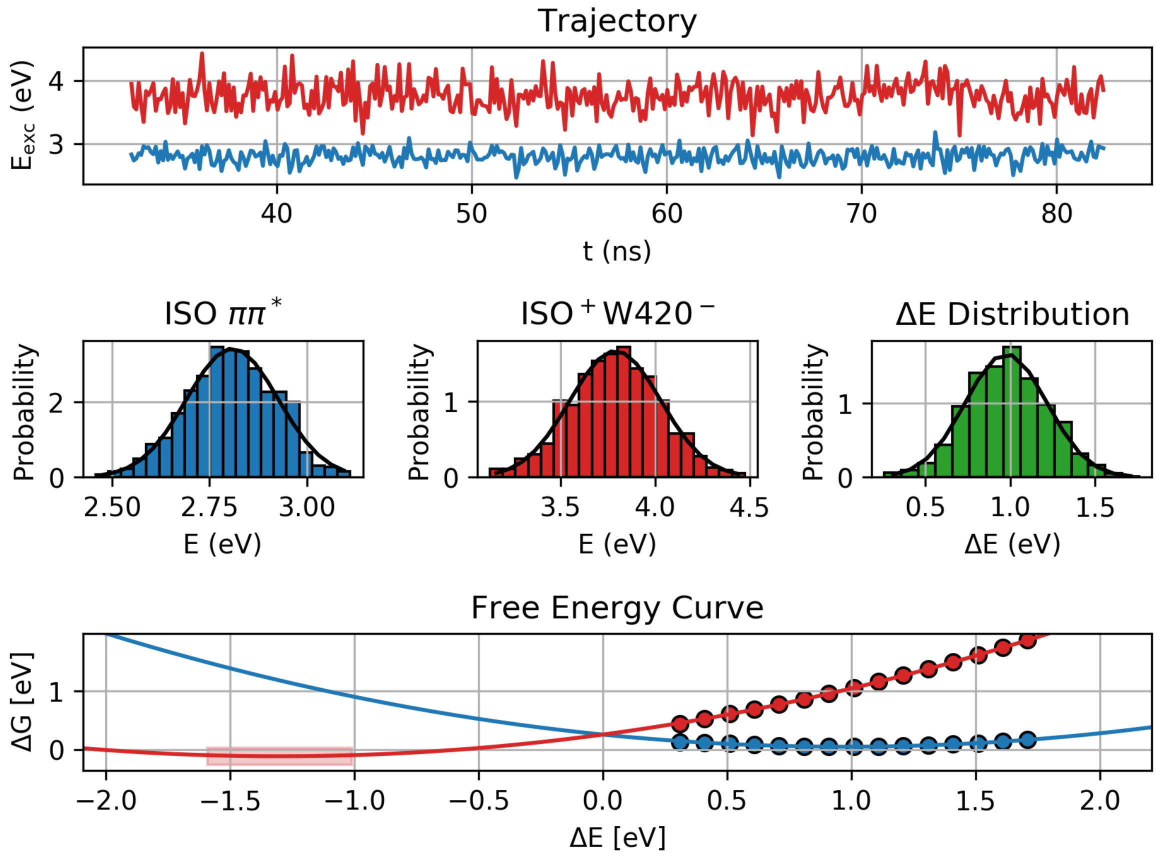

Figure S4. Free energy driving force ΔG of charge separates states: fluctuation of excitation energy E_{exc} of the $\pi\pi^*$ state and CT state ISO^-W420^+ along trajectory interval I [32.4 - 82.8] ns of the ground state equilibrium MD trajectory (top); Histograms of excitations energies of respective electronic states together with energy differences ΔE and Gaussian fits for configurations of intervals I [32.4 - 82.8] ns and II [190.8 - 241.2] ns of the ground state equilibrium MD trajectory ($\approx$ 800 snapshots, cf. Table S2) evaluated on QM(TD-DFT(BNL))/MM(ff14SB) level of theory (middle); Parabolic fit of free energy curves $G_n(\Delta E)$ (Eq. 4-5) with 95% confidence interval indicated in red.

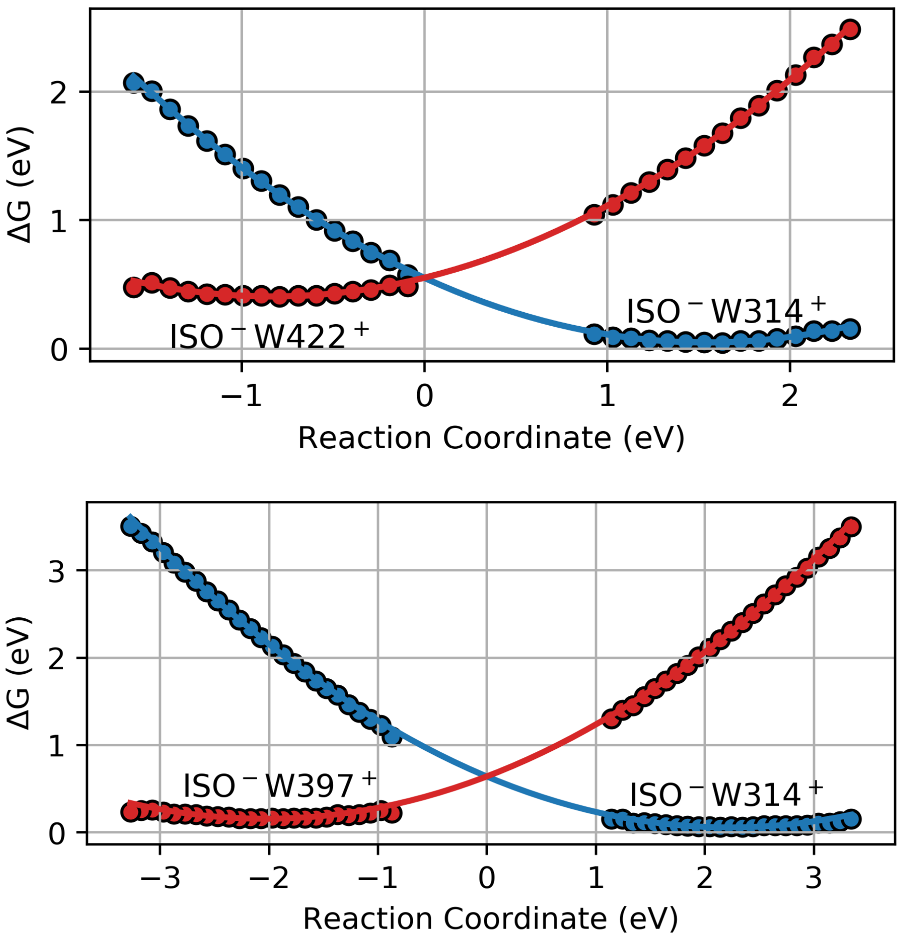

Figure S5. Free energy driving force ΔG of charge separates states: bi-parabolic fit of free energy curves $G_n(\Delta E)$ (Eq. 4-5) of CT self exchange reactions $ISO^{-}W314W422^{+} \rightarrow ISO^{-}\ W314^{+}W422$ (top) and $ISO^{-}W314W397^{+} \rightarrow ISO^{-}\ W314^{+}W397$ (bottom), for details see text.

IV. PROTEIN ENVIRONMENT ELECTROSTATIC CONTROL

In order to investigate the influence of the non-anisotropic protein environment on CT state energetics a systematic evaluation of surrounding amino acid residues was performed. 121 residues within 10 Å of the FAD cofactor were selected and the point charges of a single amino acid residue were removed from the dCRY point charge field polarizing the QM region. QM(TD-DFT(BNL))/MM(*ff14SB*) simulation of $\pi\pi^*$, ISO$^-$W397$^+$, ISO$^-$W420$^+$, ISO$^-$ADE$^+$ and ISO$^-$W314$^+$ state energies (cf. Sec II) were performed for representative geometry of the most populated cluster obtained from advanced cluster analysis (cf. Sec I). Impact ΔE of single amino acid residues was quantified as deviation from the ISO$^-$W420$^+$ and ISO$^-$W314$^+$ CT state energy with the entire dCRY point charge field (QM(TD-DFT(BNL))/MM(*ff14SB*) reference energies ISO$^-$W420$^+$: 4.091 eV; ISO$^-$W314$^+$: 4.610 eV). Fig. 3 of the main manuscript summarizes the results for amino acids that have a strong influence, affecting respective CT states by > 0.2 eV with positive values denoting destabilization and negative values denoting stabilization of the respective CT state. $\Delta\Delta E$ denotes the difference stabilization/destabilization ΔE of CT states ISO$^-$W420$^+$ and ISO$^-$W314$^+$ with negative values favoring ISO$^-$W314$^+$ and positive values favoring ISO$^-$W420$^+$

Figure S6 provides an overview over spatial arrangement of predicted key amino acid residues. We find that amino acid residues affecting both, the ISO$^-$W420$^+$ and ISO$^-$W314$^+$ CT states similarly are located near the FAD cofactor. The electrostatic interaction with the generated negative charge at the ISO moiety leads to destabilization of CT states for negatively charged residues (D412 and D410) and stabilization of CT states by positively charged residues (R381). Residues affecting CT states ISO$^-$W420$^+$ and ISO$^-$W314$^+$ in opposite direction are E398 and R298. The negative charge of E398 stabilizes the positive charge at W420 of the Trp-triad. In contrast, the positive charge of R298, located in vicinity of W314 leads to a destabilization of ISO$^-$W314$^+$ CT state. Together both amino acids favor CT pathways towards the Trp-triad due to generated intra-protein electrostatic field aligned in direction with the FAD cofactor and W420 and W397. Similarly E530 of the CTT stabilizes the generated positive charge of the ISO$^-$W314$^+$ and ISO$^-$W422$^+$ CT states.

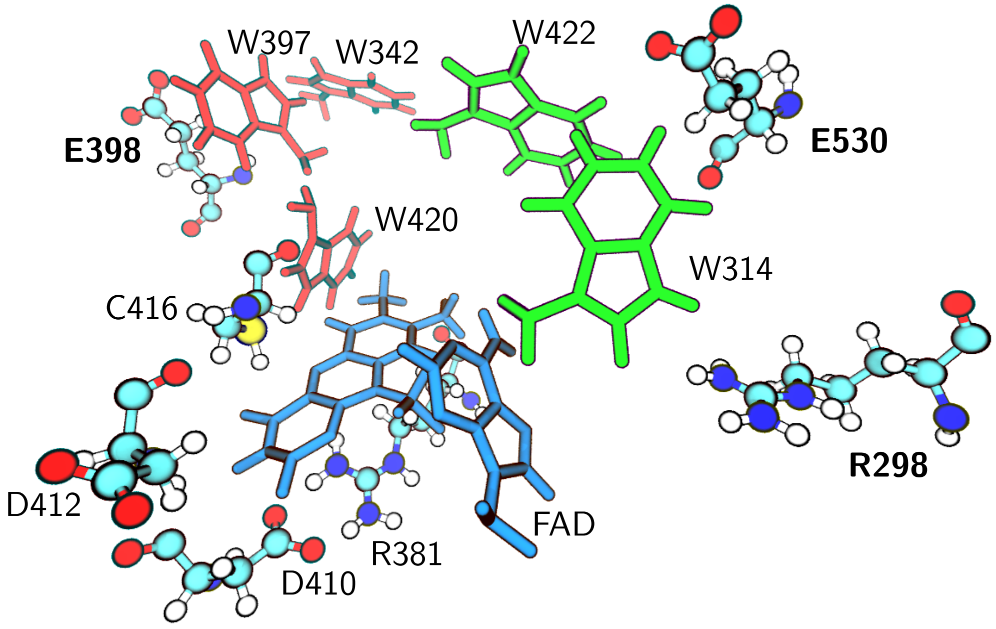

Figure S6. Spacial arrangement of FAD cofactor (blue) and tryptophan residues W420, W397 and W342 of the Trp-triad (red), as well as W314 and W422 proximate to CTT (green). Additionally, amino acid residues are shown that substantially affect $ISO^{-}W420^{+}$ and $ISO^{-}W314^{+}$.

V. MODEL HAMILTONIAN

A. Wild-type dCRY

Employing free energy driving forces ΔG^{ct} and reorganization energies λ^{ct} summarized in Table 1 (main text) the model Hamiltonian of dCRY was constructed by accounting for the systematic error of QM(TD-DFT(BNL))/MM(*ff14SB*) method compared to QM(LCC2)/MM(*ff14SB*) (cf. Table S1). Interstate electronic couplings V_{ij} were calculated with the fragment-charge difference (FCD) method[17] for configurations evaluated along the equilibrium trajectory (median of $\approx$ 800 snapshots, 50.4 ns periods I and II, cf Fig. 1 of main text and Table S2). We note that the FCD method provided more reliable results than a generalized Mulliken-Hush (GMH) treatment, in particular for situations with pronounced state mixing between CT and locally excited states.

By introducing the ISO located and initially excited $\pi\pi^*$ state located as energy origin, the Hamiltonian of wildtype dCRY accounts for CT states of the Trp-triad ($\text{ISO}^-\text{W420}^+$ and $\text{ISO}^-\text{W397}^+$) and further takes into account CT states proximate to the CTT (ISO^-ADE^+ and $\text{ISO}^-\text{W314}^+$) avoiding pre-bias towards either CT pathway. Both CT pathways account for nearest neighbor couplings (e.g. V_{12}) as well as long range CT (e.g. V_{13}), characterized by substantially reduced couplings (< 30 cm^{-1}). The system Hamiltonian is given (in cm^{-1}) by

$$H = \begin{pmatrix} \text{ISO}^-\text{W397}^+ & V_{12} & V_{13} & 0 & 0 \\ V_{21} & \text{ISO}^-\text{W420}^+ & V_{23} & 0 & 0 \\ V_{31} & V_{32} & \pi\pi^* & V_{34} & V_{35} \\ 0 & 0 & V_{43} & \text{ISO}^-\text{ADE}^+ & V_{45} \\ 0 & 0 & V_{53} & V_{45} & \text{ISO}^-\text{W314}^+ \end{pmatrix}$$

$$= \begin{pmatrix} -13085 & 337 & 15 & 0 & 0 \\ 337 & -2473 & 59 & 0 & 0 \\ 15 & 59 & 0 & 101 & 26 \\ 0 & 0 & 101 & -1127 & 117 \\ 0 & 0 & 26 & 117 & -15796 \end{pmatrix} \quad (8)$$

The Hamiltonian in eV (couplings V_{ij} in meV for readability) is:

$$H = \begin{pmatrix} -1.622 & 0 & 0 & 0 & 0 \\ 0 & -0.307 & 0 & 0 & 0 \\ 0 & 0 & 0 & 0 & 0 \\ 0 & 0 & 0 & -0.140 & 0 \\ 0 & 0 & 0 & 0 & -1.958 \end{pmatrix} + \begin{pmatrix} 0 & 41.8 & 1.9 & 0 & 0 \\ 41.8 & 0 & 7.3 & 0 & 0 \\ 1.9 & 7.3 & 0 & 12.5 & 3.2 \\ 0 & 0 & 12.5 & 0 & 14.5 \\ 0 & 0 & 3.2 & 14.5 & 0 \end{pmatrix} \cdot 10^{-3} \tag{9}$$

B. *In-silicio* dCRY mutant

Amino acid residues with profound influence on relative energetics of CT states within the electrostatic environment of dCRY (cf. Sec. IV and Fig. 3 of main text) are R298 and E398 which are suggested as key residues that have stabilizing effect on the energetics of Trp-triad CT states (see also Fig. S5). *In-silicio* dCRY mutant was generated by re-evaluating QM(TD-DFT(BNL))/MM(*ff14SB*) excitation energies and electronic couplings along the ground state equilibrium trajectory (400 snapshots, 50.4 ns period I, cf. Fig. 1 of main text and Table S2) upon neutralization of point charges of the guanidinium group of R298 (NH-C-$(NH_2)_2^+$), 9 atoms) and the carboxyl group of E398 (COO^-, 3 atoms). This treatment accounts for shifts of CT states due to the modified electrostatic environment but neglects effects on structural dCRY dynamics due to modified side chains apparent in *in vivo / in vitro* mutations of respective amino acids. Moreover we assume that the magnitude of reorganization energies is preserved upon *in-silicio* dCRY mutation. The *in-silicio* mutant dCRY Hamiltonian is derived from the wildtype dCRY Hamiltonian (Eq. 8) by accounting for respective energetic shift of the vertical average excitation energy ΔE_{exc} and correcting for systematic energy shifts of the QM(TD- DFT(BNL))/MM(ff14SB) and QM(LCC2)/MM(ff14SB) treatment (cf. Table S1). The *in-silicio* mutant dCRY Hamiltonian reads (in cm^{-1}):

$$H = \begin{pmatrix} -4073 & 305 & 7 & 0 & 0 \\ 305 & 1898 & 43 & 0 & 0 \\ 7 & 43 & -178 & 106 & 19 \\ 0 & 0 & 106 & -5508 & 131 \\ 0 & 0 & 19 & 131 & -22312 \end{pmatrix} \tag{10}$$

The Hamiltonian in eV (couplings V_{ij} in meV for readability) is:

$$H = \begin{pmatrix} -0.505 & 0 & 0 & 0 & 0 \\ 0 & 0.235 & 0 & 0 & 0 \\ 0 & 0 & -0.022 & 0 & 0 \\ 0 & 0 & 0 & -0.683 & 0 \\ 0 & 0 & 0 & 0 & -2.766 \end{pmatrix} + \begin{pmatrix} 0 & 37.8 & 0.9 & 0 & 0 \\ 37.8 & 0 & 5.3 & 0 & 0 \\ 0.9 & 5.3 & 0 & 13.1 & 2.4 \\ 0 & 0 & 13.1 & 0 & 16.2 \\ 0 & 0 & 2.4 & 16.2 & 0 \end{pmatrix} \cdot 10^{-3} \quad (11)$$

C. CTT-deficient dCRYΔ

In order to asses the influence of the CTT domain on CT energetics, a Δ-deficient *in silicio* variant dCRYΔ was constructed by neutralizing charges of terminal 20 amino acid residues (P520 to D539) followed by a re-evaluation of QM(TD-DFT(BNL))/MM(*ff14SB*) excitation energies and electronic couplings along the ground state equilibrium trajectory (400 snapshots, 50.4 ns period I, cf. Fig. 1 of main text and Table S2). In particular negatively charged E530 proximate to W314 and W422 was identified to substantially stabilize CT states proximate to the CTT relative to the Trp-triad pathway (cf. Fig. 3 of main text). The resulting Hamiltonian (accounting for QM(TD- DFT(BNL))/MM(ff14SB) and QM(LCC2)/MM(ff14SB) systematic corrections (cf. Table S1) and employing wild-type dCRY reorganization energies) is constructed form derived vertical average excitation energies ΔE_{exc} and is given by (in cm^{-1}):

$$H = \begin{pmatrix} -17830 & 287 & 21 & 0 & 0 \\ 287 & -4176 & 59 & 0 & 0 \\ 21 & 59 & 255 & 104 & 38 \\ 0 & 0 & 104 & 2114 & 147 \\ 0 & 0 & 38 & 147 & -7778 \end{pmatrix} \quad (12)$$

The Hamiltonian in eV (couplings V_{ij}in meV for readability) is:

$$H = \begin{pmatrix} -2.211 & 0 & 0 & 0 & 0 \\ 0 & -0.518 & 0 & 0 & 0 \\ 0 & 0 & 0.032 & 0 & 0 \\ 0 & 0 & 0 & 0.262 & 0 \\ 0 & 0 & 0 & 0 & -0.964 \end{pmatrix} + \begin{pmatrix} 0 & 35.6 & 2.6 & 0 & 0 \\ 35.6 & 0 & 7.3 & 0 & 0 \\ 2.6 & 7.3 & 0 & 12.9 & 4.7 \\ 0 & 0 & 12.9 & 0 & 18.2 \\ 0 & 0 & 4.7 & 18.2 & 0 \end{pmatrix} \cdot 10^{-3} \quad (13)$$

We note that the Δ-deficient *in silicio* variant dCRYΔ serves for the investigation of CTT electrostatics on CT dynamics and is not intended to fully resemble dCRYΔ[28,29] where the PHR domain of dCRY constitutes the relevant domain for light detection, whereas the CTT acts as light-dependent suppressor affecting e.g. CRY-TIM interaction.

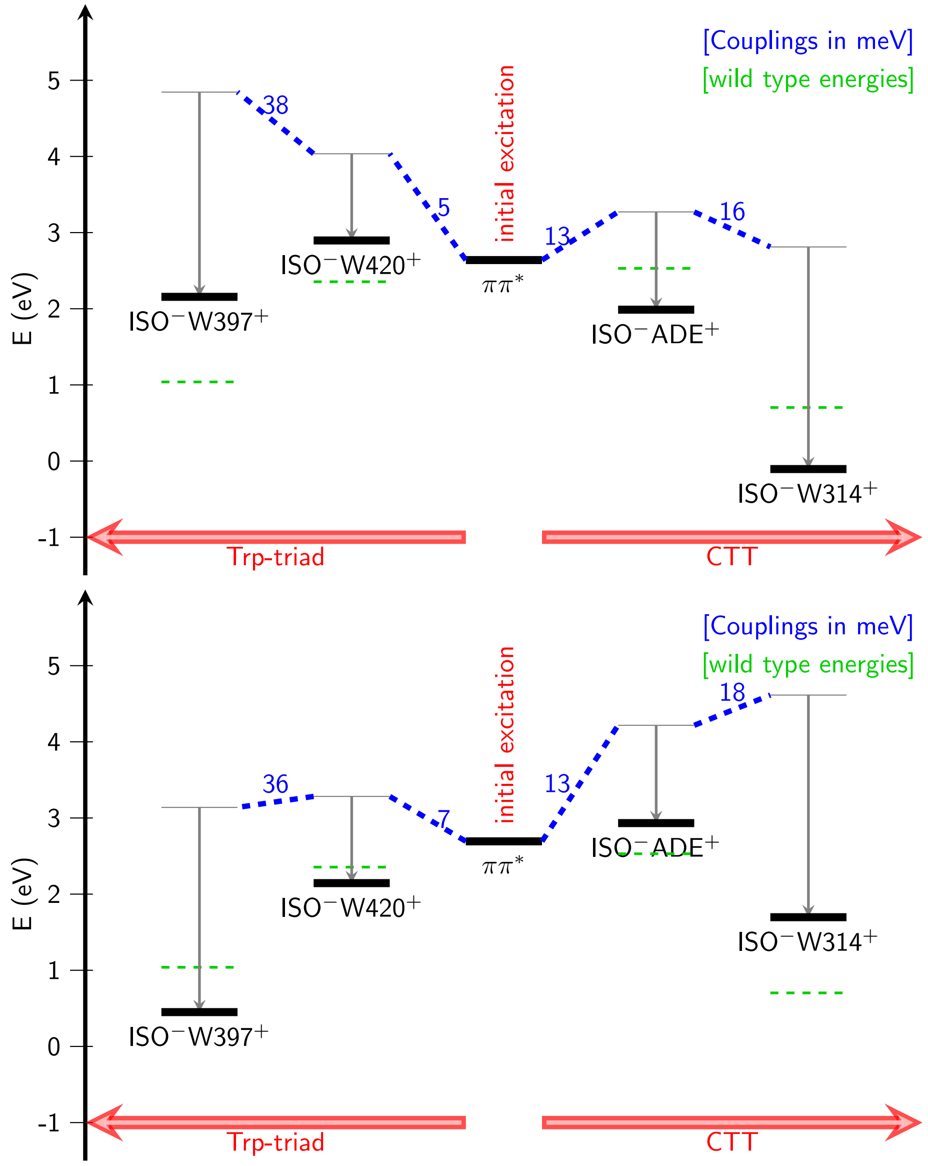

Figure S7. Jablonski diagram of state energies of *in-silicio* dCRY mutant (top) and Δ-deficient variant dCRYΔ (bottom) together with interstate electronic couplings V_{ij}.

VI. MACGIC-QUAPI DYNAMICS:

A. Spectral Density:

Details of shape and amplitude of the spectral density define the microscopic details of the nuclear bath coordinates of the environment and associated reorganization energy, and affect dynamics in MACGIC-QUAPI[30] simulations. The spectral density of ISO $\pi\pi^*$ state, CT state ISO$^-$ADE$^+$ and CT state ISO$^-$W422$^+$ (Fig. S8) was numerically calculated by Fourier transform of the energy gap fluctuation correlation function

$$J(\omega) = \frac{\omega}{k_\mathrm{B}T}\int_{-\infty}^{+\infty} \langle \delta\Delta E(0)\delta\Delta E(t)\rangle e^{i\omega t}\mathrm{d}t \tag{14}$$

employing QM/MM excitation energies (Sec. II) along a 40 ps segment of the ground state equilibrium trajectory with a sampling rate of 10 fs. Excited state energies of the 4000 snapshots (cf. Table S2) were evaluated on QM(TD- DFT(BNL))/MM(ff14SB) level of theory considering 10 singlet states.

Eq. 14 provides an alternative access to the reorganization energy via

$$\lambda^J = \frac{1}{\pi}\int_0^\infty \frac{J(\omega)}{\omega}\mathrm{d}\omega = \frac{1}{\pi k_\mathrm{B}T}\int_0^\infty \left[\int_{-\infty}^{+\infty} \langle \delta\Delta E(0)\delta\Delta E(t)\rangle e^{i\omega t}\mathrm{d}t\right]\mathrm{d}\omega. \tag{15}$$

Such treatment is limited to residues explicitly accounted for in the QM region for the evaluation of $J(\omega)$ (ISO, ADE and W422). Calculated reorganization energies are 0.42 eV for the ISO $\pi\pi^*$ state, 1.44 eV for ISO$^-$ADE$^+$ CT state and 2.06 eV for the ISO$^-$W422$^+$ CT state, in fair agreement with values derived from Eqs. 1,2 and 7 (cf. Table 1 in main text).

Direct use of the resulting spectral density in MACGIC-QUAPI simulations requires infeasible long memory time due to the peaked nature of the spectral density and the large energy spectrum of the Hamiltonian (Eqs. 8-12). Expressing the spectral density in the form of a N-peak Drude-Lorentz function of the form

$$J_N^\mathrm{DL}(\omega) = \sum_{k=1}^{N}\left(\frac{\nu_k\lambda_k\omega}{\nu_k^2 + (\omega+\Omega_k)^2} + \frac{\nu_k\lambda_k\omega}{\nu_k^2 + (\omega-\Omega_k)^2}\right) \tag{16}$$

(exact for $N \to \infty$) provides access to approximate forms by restricting the summation to lower orders. Evaluation of the reorganization energy via the multi-peak reorganization energies λ_k of the Drude-Lorentz fit allow to test convergence of the approximate treatment, parameters of single- and double peak Drude-Lorentz fit functions are summarized in Table S3.

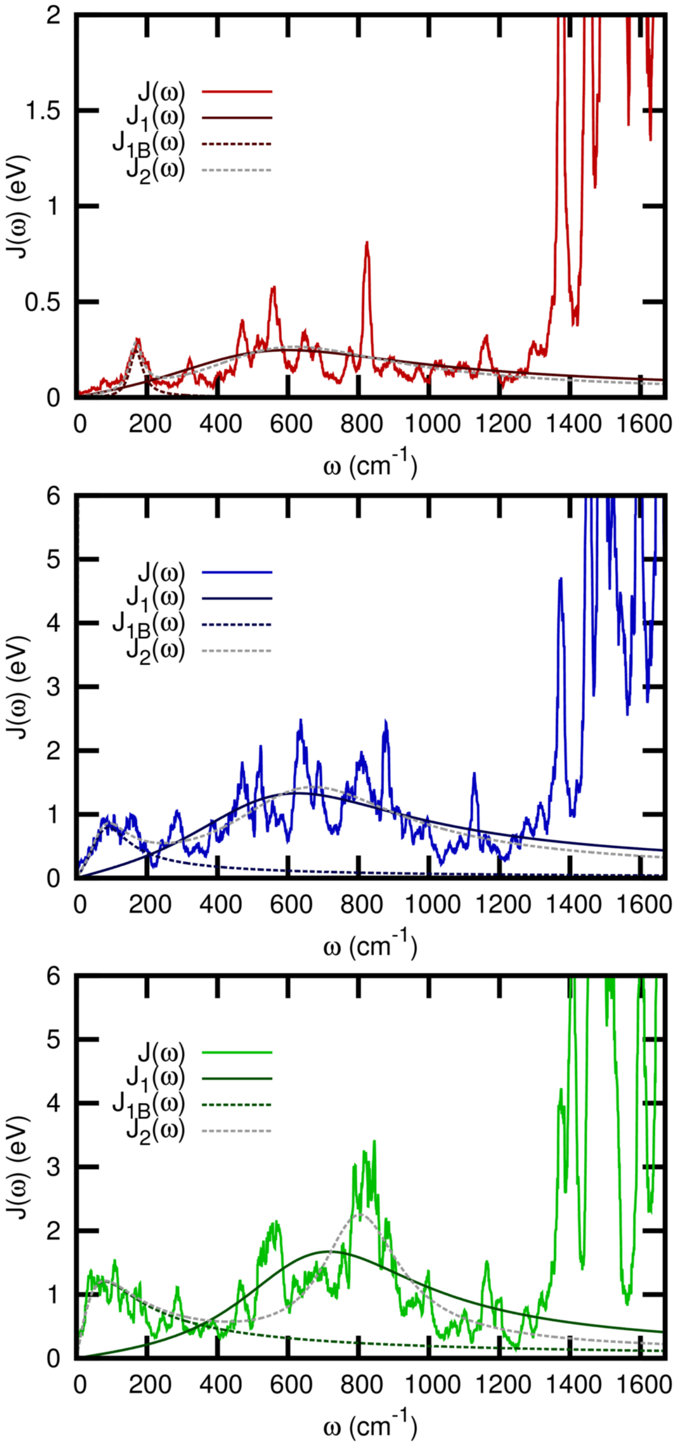

Figure S8. Spectral densities $J(\omega)$ of ISO $\pi\pi^*$ (top), ISO$^-$ADE$^+$ (middle) and ISO$^-$W422$^+$ (bottom) with fitted Drude-Lorentz functions employing one ($J_1(\omega)$) and two ($J_2(\omega)$) peaks. $J_{1B}(\omega)$ depicts the low frequency part of the dual peak fits.

Table S3. Reorganization energies λ^J (in eV) and parameters of multi-peak Drude-Lorentz functions depicted in Fig S8:

$\pi\pi^*$	λ_k (eV)	ν_k (cm^{-1})	Ω_k (cm^{-1})
λ^J 0.42			
λ_1^{DL} 0.17	0.17	402	444
λ_2^{DL} 0.17	0.14	327	528
	0.03	22	168
**ISO^-ADE^+**	λ_k (eV)	ν_k (cm^{-1})	Ω_k (cm^{-1})
λ^J 1.44			
λ_1^{DL} 0.79	0.79	375	503
λ_2^{DL} 1.11	0.57	292	611
	0.54	62	66
**ISO^-W422^+**	λ_k (eV)	ν_k (cm^{-1})	Ω_k (cm^{-1})
λ^J 2.06			
λ_1^{DL} 0.74	0.74	316	644
λ_2^{DL} 1.53	0.33	133	794
	1.20	80	0

B. MACGIC-QUAPI Dynamics and Convergence

In performed MACGIC-QUAPI[30] simulations each state was coupled to its own independent heat bath characterized by the shape of the single peak Drude-Lorentz spectral density $J_1(\omega)$ shown in Fig. S8 for the ISO $\pi\pi^*$ state, ISO^-ADE^+ and ISO^-W420+ states, respectively. For the ISO^-ADE^+ state, the area of $J_1(\omega)$ was rescaled to $\lambda^{\Delta E} = 1.28$ eV, whereas for the different ISO^-W^+ states the shape of $J_1(\omega)$ of the ISO^-W422^+ CT state was employed and scaled to respective λ^{ct} (cf. Sec. III and Table 1 of main text). The reorganization energy for ISO $\pi\pi^*$ is set to 0.01 eV neglecting limited reorganization of the $\pi\pi^*$ state.[11,27] Respective dynamics of wildtype dCRY, *in-silico* mutant dCRY and CRYΔ variant model Hamiltonians is shown in Fig. 4 of the main text, parameters of the MACGIC-QUAPI simulations are summarized in Table S4.

Table S4. Parameters of MACGIC-QUAPI simulations: M - number of states. $t_{\max}$ - propagation time , Δt - propagation timestep, τ_M -memory time, $\Delta k_{\max}$ - path length, k_{eff} - mask size, θ - filter cutoff threshold; further, the number of conceivable paths for mask size ($M^{2k_{\text{eff}}}$) and effectively used number of paths in propagation (L_{MACGIC}) is given. CPU time per propagation step on 20 core Intel Xeon E5-2640 v4 processor.

M	$t_{\max}$ (ps)	Δt (fs)	τ_M (fs)	$\Delta k_{\max}$	k_{eff}	θ	$M^{2k_{\text{eff}}}$	L_{MACGIC}	CPU Time (s/step)	Figure
5	75	0.042	43.4	1024	48	10^{-8}	$1.26 \cdot 10^{67}$	$4.47 \cdot 10^4$	0.415	4a, S9
5	99	0.035	36.0	1024	48	10^{-8}	$1.26 \cdot 10^{67}$	$3.36 \cdot 10^4$	0.279	4b, S10
5	75	0.041	41.9	1024	48	10^{-8}	$1.26 \cdot 10^{67}$	$4.52 \cdot 10^4$	0.253	4c
5	75	0.042	43.4	1024	64	10^{-9}	$1.26 \cdot 10^{67}$	$3.01 \cdot 10^5$	11.606	S9
3	10	0.073	149.8	2048	104	10^{-7}	$1.74 \cdot 10^{99}$	$9.74 \cdot 10^3$	0.502	S10
3	10	0.073	299.7	4096	80	10^{-7}	$2.18 \cdot 10^{76}$	$6.15 \cdot 10^3$	0.523	S10

Convergence of employed parameters in MACGIC-QUAPI simulations, i.e., propagation time step Δt, memory time τ_M, path selection threshold θ and the size of auxiliary merging mask k_{eff} was further analyzed. Δt is defined by the energy spectrum of the Hamiltonian where due to large energy scale of CT reactions small step size $\Delta t < 0.042$ fs is necessary. Memory time τ_M is set by the correlation time of the environment where for the employed single-peak Drude-Lorentz spectral density $J_1(\omega)$ a 40 fs correlation time is found, corresponding to a path length $\Delta k_{\max} = 1024$. For the dual peak Drude-Lorentz spectral

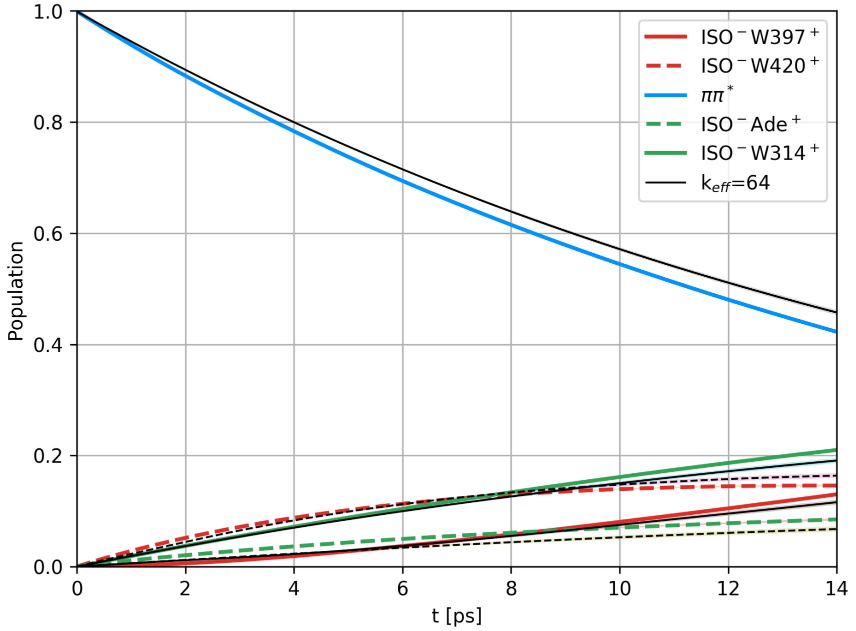

Figure S9. Convergence of mask size k_{eff} for wildtype dCRY: simulations employed a single-peak Drude-Lorentz spectral density$J_1(\omega)$, $\Delta k_{\max} = 1024$ and $k_{\mathrm{eff}} = 48$ (colors). The simulated dynamics agrees with dynamics employing an increased mask size $k_{\mathrm{eff}} = 64$ (black). See Table S4 for parameters.

density $J_2(\omega)$ (Fig. S8 and Table S3) memory time is about $\tau_M \approx 170$ fs, corresponding to $\Delta k_{\max} = 4096$ time steps (see below and Fig S10). Long time dynamics (75-99 ps) presented in Fig. 4 of the main text have been performed with tight path selection threshold $\theta = 10^{-8}$ to assure norm conservation, while short time dynamics (< 10 ps) can be efficiently performed with reduced threshold $\theta = 10^{-7}$. Convergence with the size of auxiliary merging mask is investigated in Fig S9 where due to large $\Delta k_{\max}$, a full QUAPI treatment ($\Delta k_{\max} = k_{\mathrm{eff}}$) is not possible for dCRY. For $J_1(\omega)$ and $\Delta k_{\max} = 1024$ in wildtype dCRY we find a convergence of population dynamics for an increase in mask size from $k_{\mathrm{eff}} = 48$ to $k_{\mathrm{eff}} = 64$ where both dynamical simulations show population branching between states of the Trp-triad (ISO$^-$W420$^+$, ISO$^-$W397$^+$) and proximate to the CTT (ISO$^-$ADE$^+$, ISO$^-$W314$^+$).

The effect of the shape of the spectral density on dynamics was further analyzed with a reduced 3-state model derived from the *in-silico* mutant of dCRY (Eq. 10) restricted to dynamics via CTT proximate CT states ISO$^-$ADE$^+$ and ISO$^-$W314$^+$ (Fig. S10). We note

that employed spectral density functions were scaled for equal reorganization energies in all simulations to asses the impact of different low- and high frequency regions on the dynamics.

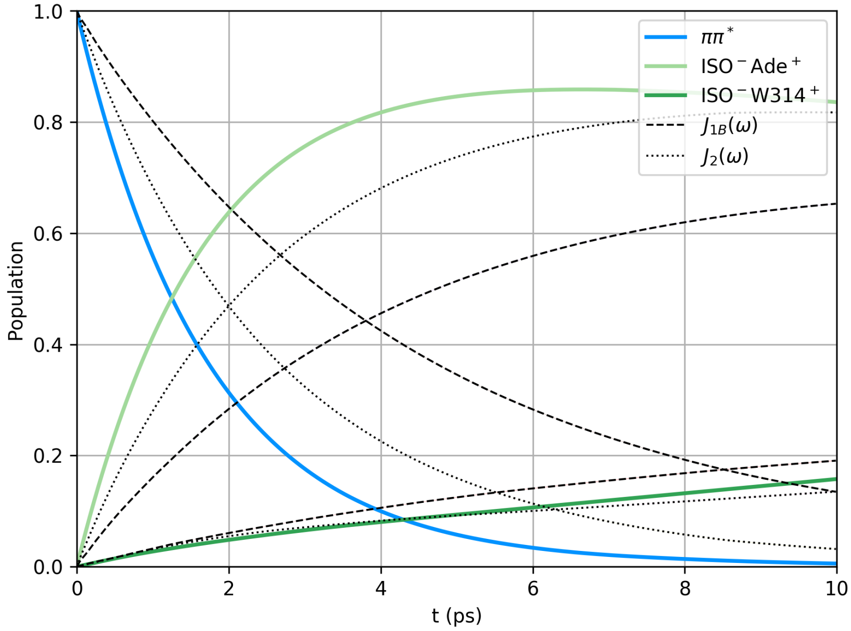

Figure S10. Effect of spectral density on CT dynamics: color lines - single peak Drude-Lorentz spectral density $J_1(\omega)$ corresponding to data of Fig. 4b of main text (*in-silico* mutant dCRY, full 5 state model); dotted lines - dynamics using dual peak Drude-Lorentz spectral density $J_2(\omega)$; dashed lines - dynamics with low frequency single peak Drude Lorentz spectral density $J_{1B}(\omega)$. Employed spectral density functions were scaled for equal reorganization energies in all simulations.

REFERENCES

[1] C. Levy, B. D. Zoltowski, A. R. Jones, A. T. Vaidya, D. Top, J. Widom, M. W. Young, N. S. Scrutton, B. R. Crane, and D. Leys, "Updated structure of drosophila cryptochrome," Nature **495**, E3–E4 (2013).

[2] J. Wang, R. M. Wolf, J. W. Caldwell, P. A. Kollman, and D. A. Case, "Development and testing of a general amber force field," Journal of Computational Chemistry **25**, 1157–1174 (2004).

[3] A. Jakalian, B. L. Bush, D. B. Jack, and C. I. Bayly, "Fast, efficient generation of high-quality atomic charges. am1-bcc model: I. method," Journal of Computational Chemistry **21**, 132–146 (2000).

[4] A. Jakalian, D. B. Jack, and C. I. Bayly, "Fast, efficient generation of high-quality atomic charges. am1-bcc model: Ii. parameterization and validation," Journal of Computational Chemistry **23**, 1623–1641 (2002).

[5] V. Hornak, R. Abel, A. Okur, B. Strockbine, A. Roitberg, and C. Simmerling, "Comparison of multiple amber force fields and development of improved protein backbone parameters," Proteins: Struct., Funct., Bioinf. **65**, 712–725 (2006).

[6] P. Li, B. P. Roberts, D. K. Chakravorty, and K. M. Merz, "Rational design of particle mesh ewald compatible lennard-jones parameters for +2 metal cations in explicit solvent," *Journal of Chemical Theory and Computation*, Journal of Chemical Theory and Computation **9**, 2733–2748 (2013).

[7] I. S. Joung and T. E. Cheatham, "Determination of alkali and halide monovalent ion parameters for use in explicitly solvated biomolecular simulations," *The Journal of Physical Chemistry B*, The Journal of Physical Chemistry B **112**, 9020–9041 (2008).

[8] D. Case, V. Babin, J. Berryman, R. Betz, Q. Cai, D. Cerutti, T. Cheatham, T. Darden, R. Duke, H. Gohlke, A. Goetz, S. Gusarov, N. Homeyer, P. Janowski, J. Kaus, I. Kolossváry, A. Kovalenko, T. Lee, S. LeGrand, T. Luchko, R. Luo, B. Madej, K. Merz, F. Paesani, D. Roe, A. Roitberg, C. Sagui, R. Salomon-Ferrer, G. Seabra, C. Simmerling, W. Smith, J. Swails, Walker, J. Wang, R. Wolf, X. Wu, and P. Kollman, *Amber 14* (University of California, San Francisco, 2014).

[9] D. R. Roe and T. E. Cheatham, "Ptraj and cpptraj: Software for processing and analysis of molecular dynamics trajectory data," *Journal of Chemical Theory and Computation*, Journal of Chemical Theory and Computation **9**, 3084–3095 (2013).

[10] M. Ester, H.-P. Kriegel, J. Sander, and X. Xu, "A density-based algorithm for discovering clusters a density-based algorithm for discovering clusters in large spatial databases with noise," in *Proceedings of the Second International Conference on Knowledge Discovery and Data Mining*, KDD'96 (AAAI Press, 1996) pp. 226–231.

[11] I. A. Solov'yov, T. Domratcheva, A. R. Moughal Shahi, and K. Schulten, "Decrypting cryptochrome: Revealing the molecular identity of the photoactivation reaction," *Journal of the American Chemical Society*, J. Am. Chem. Soc. **134**, 18046–18052 (2012).

[12] R. Baer and D. Neuhauser, "Density functional theory with correct long-range asymptotic behavior," Phys. Rev. Lett. **94**, 043002 (2005).

[13] E. Livshits and R. Baer, "A well-tempered density functional theory of electrons in

molecules," Physical Chemistry Chemical Physics **9**, 2932–2941 (2007).

[14]T. Stein, L. Kronik, and R. Baer, "Reliable prediction of charge transfer excitations in molecular complexes using time-dependent density functional theory," *Journal of the American Chemical Society*, Journal of the American Chemical Society **131**, 2818–2820 (2009).

[15]R. Baer, E. Livshits, and U. Salzner, "Tuned range-separated hybrids in density functional theory," *Annual Review of Physical Chemistry*, Annual Review of Physical Chemistry **61**, 85–109 (2010).

[16]Y. Shao, Z. Gan, E. Epifanovsky, A. T. B. Gilbert, M. Wormit, J. Kussmann, A. W. Lange, A. Behn, J. Deng, X. Feng, D. Ghosh, M. Goldey, P. R. Horn, L. D. Jacobson, I. Kaliman, R. Z. Khaliullin, T. Kuś, A. Landau, J. Liu, E. I. Proynov, Y. M. Rhee, R. M. Richard, M. A. Rohrdanz, R. P. Steele, E. J. Sundstrom, H. L. Woodcock, P. M. Zimmerman, D. Zuev, B. Albrecht, E. Alguire, B. Austin, G. J. O. Beran, Y. A. Bernard, E. Berquist, K. Brandhorst, K. B. Bravaya, S. T. Brown, D. Casanova, C.-M. Chang, Y. Chen, S. H. Chien, K. D. Closser, D. L. Crittenden, M. Diedenhofen, R. A. DiStasio, H. Do, A. D. Dutoi, R. G. Edgar, S. Fatehi, L. Fusti-Molnar, A. Ghysels, A. Golubeva-Zadorozhnaya, J. Gomes, M. W. D. Hanson-Heine, P. H. P. Harbach, A. W. Hauser, E. G. Hohenstein, Z. C. Holden, T.-C. Jagau, H. Ji, B. Kaduk, K. Khistyaev, J. Kim, J. Kim, R. A. King, P. Klunzinger, D. Kosenkov, T. Kowalczyk, C. M. Krauter, K. U. Lao, A. D. Laurent, K. V. Lawler, S. V. Levchenko, C. Y. Lin, F. Liu, E. Livshits, R. C. Lochan, A. Luenser, P. Manohar, S. F. Manzer, S.-P. Mao, N. Mardirossian, A. V. Marenich, S. A. Maurer, N. J. Mayhall, E. Neuscamman, C. M. Oana, R. Olivares-Amaya, D. P. O'Neill, J. A. Parkhill, T. M. Perrine, R. Peverati, A. Prociuk, D. R. Rehn, E. Rosta, N. J. Russ, S. M. Sharada, S. Sharma, D. W. Small, A. Sodt, T. Stein, D. Stück, Y.-C. Su, A. J. W. Thom, T. Tsuchimochi, V. Vanovschi, L. Vogt, O. Vydrov, T. Wang, M. A. Watson, J. Wenzel, A. White, C. F. Williams, J. Yang, S. Yeganeh, S. R. Yost, Z.-Q. You, I. Y. Zhang, X. Zhang, Y. Zhao, B. R. Brooks, G. K. L. Chan, D. M. Chipman, C. J. Cramer, W. A. Goddard, M. S. Gordon, W. J. Hehre, A. Klamt, H. F. Schaefer, M. W. Schmidt, C. D. Sherrill, D. G. Truhlar, A. Warshel, X. Xu, A. Aspuru-Guzik, R. Baer, A. T. Bell, N. A. Besley, J.-D. Chai, A. Dreuw, B. D. Dunietz, T. R. Furlani, S. R. Gwaltney, C.-P. Hsu, Y. Jung, J. Kong, D. S. Lambrecht, W. Liang, C. Ochsenfeld, V. A. Rassolov, L. V. Slipchenko, J. E. Subotnik, T. Van Voorhis, J. M. Herbert, A. I. Krylov, P. M. W. Gill,

and M. Head-Gordon, "Advances in molecular quantum chemistry contained in the q-chem 4 program package," *Molecular Physics*, Mol. Phys. **113**, 184–215 (2015).

[17]A. A. Voityuk and N. Rösch, "Fragment charge difference method for estimating donor–acceptor electronic coupling: Application to dna π-stacks," *The Journal of Chemical Physics*, The Journal of Chemical Physics **117**, 5607–5616 (2002).

[18]D. Kats, T. Korona, and M. Schütz, "Local cc2 electronic excitation energies for large molecules with density fitting," *The Journal of Chemical Physics*, J. Chem. Phys. **125**, 104106 (2006).

[19]D. Kats, T. Korona, and M. Schütz, "Transition strengths and first-order properties of excited states from local coupled cluster cc2 response theory with density fitting," *The Journal of Chemical Physics*, The Journal of Chemical Physics **127**, 064107 (2007).

[20]D. Kats and M. Schütz, "A multistate local coupled cluster cc2 response method based on the laplace transform," *The Journal of Chemical Physics*, The Journal of Chemical Physics **131**, 124117 (2009).

[21]K. Danylo and S. Martin, "Local time-dependent coupled cluster response for properties of excited states in large molecules," *Zeitschrift für Physikalische Chemie*, Zeitung für Physicalische Chemie **224**, 601 (2010).

[22]H.-J. Werner, P. J. Knowles, G. Knizia, F. R. Manby, M. Schütz, P. Celani, W. Györffy, D. Kats, T. Korona, R. Lindh, A. Mitrushenkov, G. Rauhut, K. R. Shamasundar, T. B. Adler, R. D. Amos, A. Bernhardsson, A. Berning, D. L. Cooper, M. J. O. Deegan, A. J. Dobbyn, F. Eckert, E. Goll, C. Hampel, A. Hesselmann, G. Hetzer, T. Hrenar, G. Jansen, C. Köppl, Y. Liu, A. W. Lloyd, R. A. Mata, A. J. May, S. J. McNicholas, W. Meyer, M. E. Mura, A. Nicklass, D. P. O'Neill, P. Palmieri, D. Peng, K. Pflüger, R. Pitzer, M. Reiher, T. Shiozaki, H. Stoll, A. J. Stone, R. Tarroni, T. Thorsteinsson, and M. Wang, "Molpro, version 2015.1, a package of ab initio programs," (2015).

[23]H.-J. Werner, P. J. Knowles, G. Knizia, F. R. Manby, and M. Schütz, "Molpro: a general–purpose quantum chemistry program package," WIREs Comput Mol Sci **2**, 242–253 (2012).

[24]N. Ozturk, C. P. Selby, Y. Annayev, D. Zhong, and A. Sancar, "Reaction mechanism of drosophila cryptochrome," Proc. Natl. Acad. Sci. USA **108**, 516–521 (2011).

[25]T. Firmino, E. Mangaud, F. Cailliez, A. Devolder, D. Mendive-Tapia, F. Gatti, C. Meier, M. Desouter-Lecomte, and A. de la Lande, "Quantum effects in ultrafast electron transfers within cryptochromes," Phys. Chem. Chem. Phys. **18**, 21442–21457 (2016).

[26]W. W. Parson, Z. T. Chu, and A. Warshel, "Reorganization energy of the initial electron-transfer step in photosynthetic bacterial reaction centers," Biophys. J. **74**, 182–191 (1998).

[27]F. Cailliez, P. Müller, M. Gallois, and A. de la Lande, "Atp binding and aspartate protonation enhance photoinduced electron transfer in plant cryptochrome," J. Am. Chem. Soc. **136**, 12974–12986 (2014).

[28]A. Busza, M. Emery-Le, M. Rosbash, and P. Emery, "Roles of the two drosophila cryptochrome structural domains in circadian photoreception," Science **304**, 1503–1506 (2004).

[29]S. Dissel, V. Codd, R. Fedic, K. J. Garner, R. Costa, C. P. Kyriacou, and E. Rosato, "A constitutively active cryptochrome in drosophila melanogaster," Nature Neuroscience **7**, 834–840 (2004).

[30]M. Richter and B. P. Fingerhut, "Coarse-grained representation of the quasi adiabatic propagator path integral for the treatment of non-markovian long-time bath memory," *The Journal of Chemical Physics*, J. Chem. Phys. **146**, 214101 (2017).
